# Dilemma-focused intervention for unipolar depression: a treatment manual

**DOI:** 10.1186/s12888-016-0947-x

**Published:** 2016-07-12

**Authors:** Guillem Feixas, Victoria Compañ

**Affiliations:** Department of Clinical Psychology and Psychobiology, Faculty of Psychology, Universitat de Barcelona, Campus Mundet, Passeig Vall Hebron, 171, Barcelona, Spain; Institute of Neurosciences, Universitat de Barcelona, Campus Mundet, Passeig Vall Hebron, 171, Barcelona, Spain

**Keywords:** Depression, Mood disorders, Psychotherapy, Constructivism, Personal construct theory, Repertory grid technique, Implicative dilemma, Therapy manual

## Abstract

**Background:**

This article introduces a new treatment protocol for depression. Based on previous research which indicated the presence of cognitive conflicts in depression, this study created an intervention manual to address these conflicts.

**Method:**

The therapy manual for depressive patients followed the guideline for inclusion in clinical trials (stage II), which has received high recognition. A preliminary version (stage I) of this manual was formulated based on other, more general dilemma-focused therapy publications, inspired by personal construct theory (PCT), and input from clinical experience. The resulting version was then applied during the 8-session format of a pilot study with patients diagnosed with major depressive disorder or dysthymia. Finally, feedback was requested from seasoned and highly respected therapists, some of whom were familiar with PCT.

**Results:**

According to the mentioned guideline, the intervention manual selected the theoretical framework, in this case PCT, to include its conceptualization of depression and resolution of dilemmas (to foster clinical improvement) as a main treatment goal. The manual was then contrasted with psychoanalytic psychotherapy, cognitive-behavior therapy (CBT), motivational interviewing (MI), and other similar approaches such as cognitive-analytic therapy and coherence therapy. Following these conceptual clarifications, the specific interventions included in the manual were defined according to both categories: their unique and essential components and those conceived as common psychotherapeutic factors. Next, the general structure and content for each session were presented. The structure consisted of seven well-defined individual sessions with an additional session, which could complement any of the former sessions to address the patient’s issues in greater depth, if needed.

**Conclusions:**

This Dilemma-Focused Intervention manual aimed to improve the treatment outcome for depression by offering an intervention that could be combined with other general approaches. At its present level of definition, it allows for inclusion in controlled trials (eg, the current RCT combining group CBT with this intervention). Thus, this manual added to the existing resources in psychotherapeutic research and practice for treatment of depression.

## Background

The present manual is a revised version of a previous intervention manual on implicative dilemmas [1]. Some aspects are here extended and modified after application in cases of different degrees of severity, as well as in a pilot study conducted with people diagnosed with unipolar depression (major depressive disorder or dysthymic disorder). These amendments aim to resolve the problems aroused during the clinical application of the previous intervention manual, as well as adapting to a specific population, in this case, people diagnosed with unipolar depression. The particular format of the manual presented here is suited for use in eight individual sessions after completion of seven group sessions of cognitive-behavioral therapy (CBT) for depression (see [2] for a full description of the study). The therapeutic process ends with a group session which addresses relapse prevention. This dilemma-focused intervention (DFI) is not a general treatment for depression but rather is conceived as a specific module within a more comprehensive treatment (this time CBT, but in future developments it could be personal construct therapy, or other approaches), in which other relevant aspects for the improvement of these patients are also addressed.

This intervention manual can be applied to adults diagnosed with unipolar depression (major depressive disorder or dysthymic disorder) and who present at least one personal dilemma, as identified through the Repertory Grid (see the section on “Symptoms/disorder assessment by the therapist” for the identification of personal dilemmas) which has been administered during the pre-therapy assessment.

For writing the manual we followed the guidelines of Carroll and Nuro [3] for stage II, with the goal of the present manual being readiness for use in randomized clinical trials (specifically in [2]). Indeed, the previous work mentioned above has served the purpose of evaluating the feasibility and preliminary efficacy of the initial versions of the manual (stage I).

### General framework

#### Overview of the approach

The theoretical foundation for the intervention described in this manual is Personal Construct Theory (PCT), proposed by G. Kelly [4], which has experienced extensive development at the theoretical, clinical and research levels in recent decades (see [5] for a review). PCT takes constructive alternativism as its epistemological basis subsequently recognized as constructivist (e.g., [6–8]); as expressed by Kelly: “we assume that all of our present interpretations of the universe are subject to revision or replacement” [4; p. 15]. Each event is constructed (interpreted) differently by each person according to their construct system, and direct access to reality is not possible. Therefore, human activity is understood as a global process of construction of meaning, and human beings as organisms whose main activity is to construct the events they encounter throughout their lives (including self, others, and their symptoms or distress). Each person is seen as a scientific layman who constructs theories to explain all their experiences, which are tested continuously. Every behavior is an experiment that validates or invalidates these personal theories. When invalidation occurs and the experiment does not confirm the previous hypothesis, the theory must be revised, thus leading to learning or change processes. A continuous process of anticipation, encounter with the event and review of expectations is thus established, in what has been called the experience cycle [6, 9, 10].

According to PCT, the cognitive system consists of personal constructs that are bipolar, as they reflect the distinctions made by the person from the perception of similarities and differences in his or her experience (for example, “hot-cold”; “friendly-unfriendly”; “depressive-cheerful”). Personal constructs are organized in a network of independent and hierarchical meanings, so that the constructs at the higher hierarchical level (superordinate constructs), many of which (core constructs) make up the sense of oneself or identity, are interconnected with other more peripheral constructs at a lower hierarchical level. From this perspective, identity gives us a sense of continuity, the experience of being ourselves regardless of the passing of time or the change of situation, as well as a feeling of uniqueness fundamental to human beings. Thus, to expect a change in core constructs may prompt threat and the person’s resistance to change in an attempt to protect their system of constructs and the continuity of their sense of personal identity.

Another of the fundamental aspects of PCT is the emphasis on the relational aspects of the construction of meanings. Even constructions related to the more intimate aspects of a person are part of a relational and (micro)cultural context. People attach particular meanings to each of their interactions with others, to the others’ actions, attitudes, etc. while at the same time constructing the notion of self and their sense of identity. Therefore, in the clinical context it is essential to explore personal meanings related to others, as well as evaluating the extent to which these meanings can be shared, supported or validated by different relational contexts in which a person moves.

PCT assumes a proactive and agentic [11] view of the human being as the regulator of his or her own motivational and emotional processes and actions, partly based on the congruence or discrepancy between the construction of self and its “ideal” (like other authors such as Carver & Scheier [12]; Cervone & Shoda [13]; Higgins [14] or Mischel & Shoda [15]). Hence, it is understandable that people encounter conflicts when having to reconcile their self-concept with their personal values in the decision-making process.

The notion of conflict is often used in Psychology to explain both psychopathology and human behaviour in general. It is worth mentioning the now classic formulations of Heider and Festinger, who posited the motivational tendency resulting from these conflicts to achieve their resolution, and that when this is not accomplished they generate a state of psychological tension. Piaget himself (e.g., [16]) proposed the term “cognitive conflict”, which in a developmental context acquires a stimulant value for the reorganization of the intellectual processes of children. Unfortunately, and despite the enormous relevance of this notion, it has received little attention in the last decades largely due to the lack of methods to provide operational and measurable definitions.

PCT considers cognitive conflicts as dilemmas which the person faces and must resolve in a manner coherent with his or her sense of personal identity. In this sense, we speak of two types of cognitive conflict: dilemmatic constructs and implicative dilemmas [17]. **Dilemmatic constructs** are those that do not offer a clear course of action. According to PCT, the person selects for him/herself the construct pole that provides a greater predictability to his construct system. In some cases, this choice is not easy and both poles have advantages and disadvantages, thus both poles become desirable and/or undesirable at the same time. In certain situations this dilemmatic construction produces a blockage in the person’s ability to act.

**Implicative dilemmas** are a type of cognitive conflict in which the symptom is associated with positive dimensions of the construction of one’s self. The desired change in a given construction (for example, to stop being “depressive” and to become a “happy” person) entails for that person, in the context of her or his system of interdependent constructs, an undesired change in another construct associated to positive characteristics of self-identity (for example, to stop being a “generous” person and to become a “selfish” one). When these implicative dilemmas occur in the cognitive system then change becomes blocked because if the person tries to move in the direction of becoming more “happy” (a desired change) she or he also feels she or he is becoming more “selfish” (an undesired change). This conflict involves the sense of personal identity and it might help to explain both the onset of clinical symptoms and the resistance to treatment. This concept, therefore, offers an explanation for “resistant” behavior in patients (for example, lack of involvement in homework assignments, poor compliance with medication) and represents an alternative response to the classical conception of the neurotic paradox or symptom function. The implicative dilemma notion refers to the **structure** of cognition and, in this sense, it could appear in people with different diagnoses (or no clinical diagnosis at all). But what is specific to each case is the **content** of the dilemma as reflected in the verbal labels of the constructs involved in it.

The Repertory Grid Technique (RGT) [18, 19] is an instrument derived from PCT which allows the operational identification of cognitive conflicts. The technique has a high utilization rate, ranging from the individual clinical assessment to business consulting (see [20]). The RGT allows for the evaluation of self-concept and cognitive structure from the person’s constructs and therefore, based on the construction made of oneself and others, including the “ideal self”, in his or her own terms. Although there are other assessment methods to identify these personal dilemmas (for example, through narratives or conversation), our group has developed a way to gauge them through the RGT [17, 21], thus providing an operational way to identify ambivalences when facing a desired change. See the section “symptom/disorder evaluation by the therapist” for a detailed description.

#### Rationale for the treatment

Starting with the notion of the human being as a constructor of meanings, it is understood that the symptoms have personal meanings which arise in the process of making sense of experience. Treatment, therefore, seeks the exploration and understanding of these meanings (and the identity conflicts they entail) as well as the elaboration of more viable alternative constructions, which generate less suffering and are coherent with their sense of identity.

Having identified with the RGT the dilemma or dilemmas to be dealt with, together with the patient, we explore his or her constructions of self, others, and the problem, to understand how they affect his or her current situation and the difficulties for change. The interventions described in this manual have as their ultimate aim this exploration of the personal meanings to contribute to the resolution of the dilemmas and to foster a more harmonious, flexible and elaborate construct system, enabling the person to function without the presence of symptoms. As mentioned above, when patients have personal dilemmas in their construct system, pushing them towards a direct change regarding symptoms would result in their “resistance” as an attempt to protect their sense of identity. Therefore, treatment must focus on the exploration of the implications of this change and on making it compatible with their identity. Thus, we recognize the person’s need for continuity in his or her sense of identity and the need for coherence of his or her actions with that identity, in spite of the suffering and malfunction that keeping that coherence may involve. By focusing therapy on the dilemmas, we obtain a better starting point to seek change, at the same time further improving the therapeutic relationship [22]. Intervention is understood as a delicate renegotiation of the personal meanings of the patient and of the positive and negative implications of the symptom or problem as they configure the dilemma, without opting for one a priori solution. The therapist adopts a facilitating role in this exploration so the person can rebuild his or her life solving their own dilemmas. Ultimately, patient and therapist should jointly generate constructions appropriate to the unique and personal demands of each patient. Therefore, the interventions described do not seek to attain a stereotyped result or previously defined outcome, rather to make compatible the patient’s desired change with the maintenance of his or her sense of identity. It is not intended to unfailingly change the symptomatic construct, but rather to resolve the personal dilemma in the terms the patient considers more suitable for him or her. Notwithstanding, the interventions do seek to reduce the level of cognitive conflict, understanding that the presence of these inconsistencies in the patient’s construct system makes it difficult to adapt to the changing demands of his or her interpersonal context.

Regarding the treatment of depression, a dilemma-focused intervention (DFI) can be particularly suitable. As mentioned above, the clinical course found in these disorders is often characterized by chronicity and/or relapse. Often a diagnosed patient has followed several treatments without success and, notwithstanding, continues to experience the symptoms of depression. All this may contribute to him or her feeling hopeless and/or the feeling of being to blame for this situation. DFI aims to offer the patient a respectful explanation of the persistence of his or her symptoms in relation to the dilemmas in his or her personal construct system. Thus, an explanation of the problems based on the “disease”, his or her deficits, or cognitive errors is avoided. We stress the need for the patient’s coherence with a cognitive system that has been constructed over a lifetime, from which a sense of identity has developed, with an associated need for continuity, which needs to be harmonized with the need for change. For this reason, DFI is ideal for the treatment of depressive disorders as it strengthens the sense of personal agency and legitimizes both the desire for change and the difficulties of achieving it.

#### Theoretical mechanism of action

The exploration of the dilemmatic meanings and the constraints the dilemma entails in the patient’s life leads him or her to the need to find a viable alternative of construction that resolves these dilemmas. The intervention does not pursue the elimination of the symptom nor a specific change of behavior, rather a breakthrough in the development of the patient’s construct system, of his or her sense of identity, so that life with the symptom becomes a less meaningful option and change becomes part of this personal evolution. Thus, the central mechanisms would be those of clarification of the dilemmatic nature of the situation and of the integration of the two sides of the dilemma. The whole approach invokes the idea that change is possible and feasible when the patient’s system of meanings is harmonized, thus leaving all the power for change on the patient’s side, a process that involves activation of both empowering and self-agency mechanisms. Although the idea that resolution of internal conflicts leads to an improvement in depression has still little evidence (in [23] we found that number of implicative dilemmas was moderately correlated to depressive symptom severity), the fact that the present therapy manual has been created will enable further studies testing this assumption.

### Conception of the problem

#### Etiological factors

The approach assumed in this manual presumes the existence of genetic, biological or social factors in the etiology and maintenance of the depressive symptoms, but focuses on how these factors are depicted in the meaning ascribed to them by the patient and how he or she manages them. Other factors relevant to the treatment of depression are discussed in the group module of the treatment.

Kelly conceives depression as an extreme constriction of the perceptual field to minimize the risk of personal invalidation [4]. When the possibility of invalidation of a core construct - related to identity - exists, the person reduces his or her own experience (for example, sleeping more hours or avoiding activities and contact with other people) in an attempt to protect that meaning, to avoid the significantly higher discomfort that would result from its loss. In people with depression this natural process has become generalized, producing in turn discomfort. Other authors in PCT provide an explanation of depressive symptoms in which the notion of personal dilemma is reflected. For example, Rowe [24] argues that people persist in their depressive symptoms because they “prefer to be good than happy”, thus establishing a false dichotomy or dilemma between being good and being happy. She also describes [25], using a version of the RGT, the case of a chronically depressed patient who faced the choice between staying depressed (linked in her system to being “human”) or changing, and turning into a “destructive” or “unpleasant” person (according to her own view). In this sense, people with depression associate negative characteristics such as insensitivity, selfishness or emotional distance with being happy, while unhappiness would be associated with positive characteristics such as sensitivity, generosity or closeness.

In a series of studies conducted by our team [26] a significant relationship was found between the presence of dilemmas and depressive symptoms. Feixas, Montesano, Erazo-Caicedo, Compañ, and Pucurull [27] found a higher proportion of depressive patients with implicative dilemmas (59 vs. 39 % in the control group). Also in this study, the level of symptoms was associated to the presence of these conflicts. In a study with a non-clinical sample (*n* = 545), Varlotta found that the presence of implicative dilemmas was significantly related to higher scores on depressive symptoms [28]. These dilemmas significantly predict the score on the scale of depression of the SCL-90-R in a linear regression. Feixas, Compañ, Montesano and Saúl [29], in a sample of patients diagnosed with unipolar depression (*n* = 81), found at least one personal dilemma (dilemmatic construct or implicative dilemma) in 90 % of these patients. Specifically, the presence of implicative dilemmas was significantly higher in the clinical sample compared to the non-clinical control sample as depicted in the final report for this study [23]. Moreover, the number of implicative dilemmas in the clinical sample tripled those of the control sample. All these results indicate that, although the presence of dilemmas is not specific to depression, it seems to play an important role in this population. However, all dilemmas found in these patients are not explicitly related to the depressive symptomatology. For example, we may find dilemmas around the desire of wanting to be more sociable, more active, punctual or any other characteristic the person wishes to modify. We start from the idea that the presence of personal dilemmas, regardless of the concrete contents of the verbal label, is related to inconsistencies or fragmentation of the cognitive system. These inconsistencies may hamper the system’s flexibility and ability to act in a satisfactory way in the interpersonal context, favoring emotional distress or suffering.

If we take into consideration the course of these disorders, we find that the dysthymic disorder has by definition a chronic course, and in the major depressive disorder relapse rates can reach 85 % after 15 years, even in those patients who have been successfully treated [30]. These data evidence the great difficulty experienced by these patients to achieve a consistent and sustained symptomatic improvement. As we saw in the first section, the presence of implicative dilemmas offers an explanation for the persistence of symptoms, as well as for the so-called “resistance” in this kind of patients. In any case, the presence of dilemmas is not postulated as the only causative or maintenance factor for depression (or any other disorder) but rather as a cognitive structure that may hinder the process of change.

#### Factors associated with behavior change

The resolution of the dilemma achieved through the reconstruction of the personal meanings entails changes in the way the person acts and feels. The intervention does not necessarily seek a predetermined change of behavior, but rather a reconstruction of the meanings associated with the symptom, so that it becomes meaningless in the patient’s construct system. By constructing the events in a non-conflictive way the person responds in a different way to a given situation; he or she moves towards their goals and personal objectives without the burden or blockage posed by internal conflicts.

#### Agent of change

PCT adopts a proactive vision of the human being. That is to say, the person does not passively respond to stimuli, nor is he or she the result of mere environmental conditioning, nor of information processing. On the contrary, humans actively construct the meanings given to each of the experiences they encounter, and, as such, patients also construct a meaning for the therapist’s interventions. In this sense, techniques are used as ways to explore and suggest tentative changes of meaning, not as tools to generate a predetermined change in behavior. Therefore, patient and therapist must participate in a significant dialogue in which the patient will reconstruct his or her own experience in less dilemmatic terms. The therapist offers a context in which to explore the dilemma with no pressure to opt for one of the possible solutions. The agent of change is always the patient and his or her ability to review their own personal meanings.

#### Symptoms/disorder assessment by the therapist

This approach to therapy focuses the assessment process on the patient’s construct system, both its content and structure. The assessment of the symptoms, risk and functioning is addressed through questionnaires such as CORE-OM [31] and other self-report instruments, and it is taken into account as an indication of therapy progress.

The technique used to assess the construct system, the RGT, takes the form of a semi-structured interview in which, from a comparison of significant people, here called “elements”, bipolar constructs are elicited (for example, “shy–sociable”; “depressed–happy”). It should be emphasized that these constructs are personal to the interviewee and are written in the grid form using the same linguistic expression the person uses. The elements always include the current self and ideal self (“The way I would like to be”). Subsequently each of the elements is rated on all of the constructs, using a 7-point Likert scale. The result of this process is a matrix of scores that reflects the assessments made by the person of the elements in accordance to his or her own constructs. This matrix can be subjected to statistical analysis to synthesize and operationalize all the provided information. In our case we use the Gridcor 4.0 program, available at www.terapiacognitiva.net/record, which provides a series of indices such as, for example, measures of self-ideal discrepancy, self-perceived social isolation, the perceived adequacy of others or polarization (for details on RGT administration and analysis, see [18, 19], and particularly [17] for looking at the specific procedure for identifying cognitive conflicts).

Focusing on the identification of personal dilemmas, dilemmatic constructs are those on which the “ideal self” element receives a rating of 4, indicating that neither of the poles of the construct is desirable (maybe that they are both desirable) for the patient. In this sense, these poles do not offer a clear course of action because neither of the poles of the construct appears as preferable to the other. An example of one such construct was “detached-selfish” as provided by one of our patients. By rating his ideal self with a 4 he indicated that both being “detached” and being “selfish” had both positive and negative implications simultaneously.

**Fig. 1:**
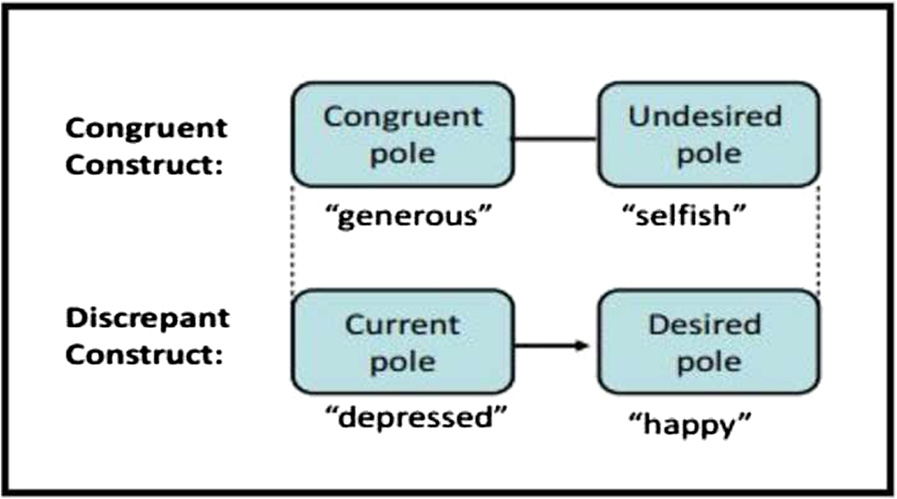
Example of an implicative dilemma

The Gridcor program provides automatic identification of the implicative dilemmas in a repertory grid. For that, it follows the following steps [17, 21]. In the first place, **congruent constructs** are identified (those in which the person rates his or her “self now” and “ideal self” similarly, that is to say, those in which he or she does not wish to change) and **discrepant constructs** (those in which the person rates his “current self” and “ideal self” at opposite poles, ie, those in which there is a desired change). We find an implicative dilemma whenever a positive correlation (*r* > 0.35) appears between the ratings of a congruent construct and those of a discrepant one, so that if a change were to happen on the discrepant construct it would result in turn in an undesirable change on the congruent construct. Figure 1 shows the basic outline of an implicative dilemma. In this example, the person being assessed describes herself as a “generous” person (versus “selfish”) and wants to remain so (to become “selfish” is not desired). She also describes herself as “depressed” and would like to be a “happy” person. However, due to the association between these two constructs, the desired change in order to become a happier person would imply an unwanted change on the other construct, feeling like a selfish person. Thus, she implicitly “decides” to maintain a positive image of herself, sacrificing her happiness for the sake of not being selfish.

#### Case formulation

The results obtained from the assessment using the RGT and the identification of personal dilemmas are the basis for the formulation of each case. Once dilemmas are identified, their practical relevance for the current problem is conveyed to the patient to focus therapeutic work in subsequent sessions.

### Treatment goals

#### Specification of the treatment goals

The overall objective of the treatment described in this manual is to reduce the discomfort associated with the depressive symptoms through the therapeutic elaboration of personal dilemmas. It is expected that this will allow the person to generate more viable and coherent alternative constructions, which should foster change in any sphere of the person’s life in the direction of significant improvement in his or her welfare. In this sense, this approach differs from others in that it does not focus on defining specific objectives for behavioral and/or symptomatic change. Although patients are encouraged to generate alternatives, therapy is not presented as a solution for specific problems.

#### Evaluation of patient’s goals

In the first session of this treatment module, patients are asked about what expectations they have for this phase of therapy. That is to say, in the first session some time is dedicated to explore the patient’s therapeutic demand [32], taking into consideration that the treatment began previously with 7 group sessions and, therefore, the patient’s demand is already outlined in psychotherapeutically approachable terms. After clarifying the patient’s expectations and their desired change in their own terms, we proceed to explore the dilemmas found through the RGT and their possible relation with the patient’s request. Following the basic tenets of PCT in regards to the therapeutic relationship (see the section “patient-therapist relationship”), application of this manual would only be possible when, after exploration of the dilemmas together with the therapist, the patient identifies it as relevant to his or her symptoms and agrees to address it in the therapy sessions. When the patient considers the presented dilemma as irrelevant or, for any other reason, is unwilling to focus on the said dilemma, the therapy will have to abandon the present protocol’s format and alternative goals can be negotiated with the patient.

#### Identification of other relevant goals

This treatment manual focuses on the elaboration of personal dilemmas. When other relevant objectives/goals exist, they are addressed only in relation to the dilemma and its manifestation in the patient’s construct system. For example, when a history of trauma exists, we explore how this has influenced the patient’s positioning regarding the dilemma.

#### Negotiation of change in goals

Throughout the whole treatment the person is encouraged to share his or her impressions with regard to the treatment and the employed techniques. The treatment objectives/goals are determined by patients, so that if they decide to leave the DFI at any time the therapist must refocus his or her efforts towards the new direction posed. However, the therapist can explore the possible relation that may exist between the new goals set by the patient and the elaboration of the personal dilemmas. For example, if the patient considers it necessary to address his or her relationship problems the therapist may explore the link between these and the presented dilemmatic construct “passive-aggressive”.

#### Contrast with other approaches

The majority of theories of psychotherapy try to explain why people do not change in spite of their desire to do so. For that, they refer to the neurotic paradox, the function of the symptom or the ambivalence to change, amongst other terms. Because of this, it is justifiable to ask oneself what the personal dilemma approach brings to psychotherapy and how it helps explain the lack of change. In this section, we will analyze the similarities and differences between the personal construct approach, and DFI as a particular development of it, and psychoanalysis, cognitive-behavioral therapy and the motivational interview. We will also do this comparison with two other related approaches: cognitive- analytic therapy and coherence therapy.

**Psychoanalysis** in its different variations and modalities is based on the notion of intrapsychic conflict. In this sense, it is similar to DFI. Furthermore, both approaches consider that the human psyche is not a unitary and logical system, but it can contain forces in conflict which may be unconscious as well as secondary gains of symptoms. Kelly [4] believed we create reality as replicas of past situations, with the same constructs, a phenomenon which he relates to transference (although he gives it a much more general sense than psychoanalysis). Regardless, the differences are substantial in the understanding of human functioning as well as in the focus of the therapy. The unconscious drive does not have a place in PCT. Although it is recognized that many constructs are non-verbal, they form a part of the same system as verbal constructs. On the other hand, the vision of PCT is basically cybernetic, understanding experience as a process in which constructions are tested out and the confirmation, or disconfirmation, of which requires revising the construct system. Therefore, it strays far from the energetic-hydraulic vision of forces in conflict of classical psychoanalysis.

The **cognitive-behavioral model** is, without a doubt, the most prestigious psychological treatment of depression. The therapeutic proposal of this approach has as its main axis the promotion of pleasant activities and the questioning of negative thoughts that automatically invade the consciousness of these patients, as well as challenging their beliefs. Dysfunctional schemas, attribution bias, rumination and perfectionism, among others, also form a part of the cognitive model of depression [33].

Because of their common cognitive focus, there are many similarities between cognitive-behavioral therapy and DFI. In fact, Kelly is often cited as a predecessor of cognitive therapies [34–36] and PCT was the first approach to make explicit the view of human beings as scientists who build theories about the self, others and the world, a common distinguishing factor of all of these therapies. From this principle the interest of designing behavioral “experiments” that test cognitions follows. The recognition that people do not respond directly to stimuli or events but to the way they are coded or interpreted is also shared. Reinforcement is considered but also the role of anticipation or expectation of what is going to happen. Both approaches assume that these constructions or schemata are organized in systems from which interpretations, inferences or predictions that people make are derived. Their role in the processes of storage and memory recall is recognized [37]. Kelly described core and peripheral constructs [4], a distinction that is paralleled by Beck et al. with the notions of core beliefs and automatic thoughts [34]. Cognitive therapies, a term that for several authors includes personal construct therapy [7, 38, 39], converge in considering change in the way people interpret their reality as a primary objective in psychotherapy. However, personal constructs were not formulated as cognitions but more holistic interpretative templates.

Certainly, the differences between CBT and DFI are also notable. Firstly, in the former, the cognitions that cause disturbance are considered cognitive distortions or maladaptive beliefs that need to be identified and modified/corrected. Regardless, in PCT the aim is not to correct logical errors. Rather, they are understood as a part of a larger system in which they have a role, and maintaining them serves a necessary purpose. The purpose of core constructs is to provide continuity in the person’s sense of identity, an essential psychological need. That is why they serve a very important role and their comprehension is necessary before starting any changes. The construct system needs to evolve in a way which maintains some continuity. Still, it can develop in a way so that the symptom loses its importance for continuity. For this goal, it is important that the therapist accepts the patient’s constructions as a part of a unique system which is necessary to make sense of reality (and the self is a very important part of it). It is useful to explore in some detail the constructions that make the symptom necessary to have, its implications, internal logic, until the conflictual nature of these constructions (in contradiction with other constructions of the patient) can be discussed and resolved.

Similarly, in assertiveness training (one of the techniques used in cognitive-behavioral therapy) a distinction is made between being “assertive” and being “aggressive” in a predominantly psychoeducational format. In contrast, in DFT the personal meanings involved in any social difficulty clients may perceive for themselves are fully explored with a focus on unrevealing the “dangers” (threats to the present sense of personal identity) which are perceived in overcoming these difficulties.

Cognitive therapy conceives the therapeutic relationship as a relationship of collaboration between patient and therapist (collaborative empiricism), although the therapist is usually directive in the structure of the sessions, in the prescription of activities and experiments, as well as the questioning of the logic and validity of the patient’s thoughts. For example, although homework is thoroughly worked out with the patient, when the patient does not perform the tasks the cognitive therapist usually defends, using data or logical arguments, the importance of their completion in order to achieve change. Thus, cognitive treatments emphasize psychoeducation in the treatment of depression model and the necessary strategies for improvement. In PCT, psychoeducation is kept to the minimal level the patient is considered the “expert” in his or her own world of meanings [6, 8]. This conception is inspired in what Kelly [4] called the “credulous attitude” of the therapist, who considered the patient’s constructions as having a “value of intrinsic truth” which should not be ignored. In DFI, homework is designed to explore personal meanings and its non-completion is taken as an opportunity to explore the meanings involved in not doing the task without insisting on these tasks. In DFI, homework assignments are oriented towards self-exploration rather than to direct improvement (asking the client to do more activities, or testing a particular belief in a behavioral experiment) or towards educating the patient in the psychological model on which the treatment is based. When trying to promote change, personal construct therapists adopt an exploratory and “invitational” [4] style and avoid criticizing the patient’s thoughts (or labeling them as negative, irrational or unrealistic), rational dispute, or demonstrating with arguments or proof that the patient’s way of thinking is wrong. Rather, the implications of his or her constructions are explored. At the time of creating alternatives, the patient is invited to consider an alternative construction and to explore its implications without necessarily adopting it or assuming that it is more realistic or correct.

In the past few years, the **motivational interview** [40, 41] has experienced noteworthy development and has been applied not only to addictive behaviors, but to a growing number of psychological problems (see [42] for a review). The motivational interview is usually defined as a directive, client-centered approach which promotes motivation to change and the ability to resolve ambivalences when faced with a certain change. According to the authors, it is especially recommended for people who are resistant to change and who show ambivalence before change. In the case of depression, the motivational interview has been used either as a prelude to psychotherapy [43] or as an integrative framework for cognitive- behavioral treatment [44–46], assuming that depressive patients tend to perceive themselves as resistant (ambivalent) and the motivational interview can increase the activity level of people with depression.

The motivational interview has some points in common with PCT, for example, its interest in attenuating the relevance of diagnostic labels in the process of change and its emphasis on personal choice and the patients’ responsibility for deciding their future course of action. On a technical level, we find some similarities as well, like the decisional balance sheet, in which advantages and disadvantages of change are explored. This table or sheet was created by Jannis and Mann [47] and is similar to the one developed by Tschudi [48], named ABC and widely used in PCT. The concept of resistance is also coincidental. In the motivational interview, resistance is an observable behavior that emerges during treatment and lets the therapist know that his or her interventions are not in tune with the patient. In spite of the similarities, the focus of the treatment presented in this manual mostly emphasizes the personal coherence that these “resistant” behaviors involve (see Table 1).

**Table 1:** Example of the Tschudi’s ABC technique applied to the construct “Passive – Active”

A:	Passive	Active
B:	*Disadvantages* You don’t achieve what you intend You become bored	*Advantages* You fight for what you want You enjoy yourself
C:	*Advantages* You make less mistakes You don’t have to think about what to do	*Disadvantages* You become more frustrated if you don’t achieve what you want You spend all day thinking what to do

The main discrepancy between both approaches lies in the conception they have of the dilemma (ambivalence) as the phenomenon which maintains the symptom. In the motivational interview, ambivalence emerges when there is an attachment to an addictive or problematic behavior. In general terms, the attachment can be due to issues of dependence and/or tolerance to a substance (for example, alcohol addiction), learning processes and to conditioning (for example, sexual deviations) or to the utilization of addictive behavior as a way of coping with difficult or unpleasant feelings (for example, to relax or to be at ease) [41]. In PCT, the difficulty for change depends on the implications of change for the sense of personal identity. Changing would imply not being who I am in some fundamental way. The person has constructed an image of himself or herself and others which is coherent with his or her experience, and the symptom is coherent with this construction; it is a course of action compatible with the person’s sense of identity. Although it produces some invalidation (negative consequences) the desired change (symptom removal) would involve a much greater invalidation of the self.

Another difference in regard to the motivational interview is the way of identifying these conflicts or ambivalences. The utilization of the RGT facilitates the detection of the dilemmas during the assessment session, and this advantage is carried forward through the therapy sessions because it involves using the patient’s own terms.

#### Similar approaches

Cognitive Analytic Therapy (CAT) is a brief integrative therapy which synthesizes theoretical and technical elements of different orientations like cognitive therapy and psychoanalysis. Like PCT, CAT considers human beings as scientists who test their hypotheses, and uses the concept of feedback and collaborative therapy. In fact, Ryle was knowledgeable both of PCT and psychoanalysis, and carried out some research with the RGT. Ryle [49] proposed three types of dysfunctional and systematically observable cognitive patterns in psychotherapy patients, which he called “traps”, “dilemmas” and “snags”. In the CAT frame, dilemmas are understood as cognitive problematic subroutines in which a significant restriction of the possible actions are produced, adopting dichotomic formulations such as “one or the other” (you are either sensible or happy) or “If I am…, therefore…” (If I am happy then I am selfish). For Ryle the identification of a precise focus of treatment with an adequate degree of abstraction for brief psychotherapy is fundamental. The dilemmas are one of these possible treatment focuses, which allow provisional hypotheses to be made between patient and therapist regarding the treatment objectives and an evaluation of the changes at the end of it. Thus, CAT shares the logic of DFI essentially because both are based in PCT. However, there are some differences on a range of issues. One of them is the way in which dilemmas are assessed. In CAT, patients respond to a questionnaire, the Psychotherapy File [50], describing a set of pre-established dilemmas. In contrast, DFI is based on the patients’ RGT, in which the content of the dilemmas is completely provided by the patient. Also, CAT uses other concepts for case formulation such as traps, snags and reciprocal roles.

Ecker and Hulley originally called their approach “Depth-Oriented Brief Therapy” [51] and more recently “**Coherence Therapy**” [52]. It is a constructivist approach that has many points in common with DFI, mainly because they share many common grounds. One of the more outstanding similarities is Ecker and Hulley’s [51] notion of the pro-symptom position as a way to explain the appearance and maintenance of a given symptom. From their perspective, the symptom has a positive value in at least one context, understanding the context as a wide conjunction of meanings attributed to situations thematically related (for example, family, romantic and social relationship, autonomy, self- esteem, etc.). Indeed, while the existence of an anti-symptom position is apparent in the patient’s presentation (eg, the patient complains of a problem or symptom which creates suffering and needs to be removed) Ecker and Hulley stress the concomitant existence of a pro-symptom position. The latter reflects the need for the symptom to maintain the patient’s personal coherence. This position is kept unconscious while the anti-symptom position is conscious so that the patient describes the symptom as completely negative, without value, and an obstacle to well-being. It is from this position that the person seeks change or goes to therapy. An objective of coherence therapy is to identify the contexts in which the symptom has a positive value and promote an elaboration in which the symptom stops making sense in the experiential reality of the patient. It is about finding an answer to the central question: “What construction makes having the symptom more important than not having it?” To respond to this question a series of different techniques, which oscillate from finishing incomplete phrases to experiential work with dreams or an examination of personal history, are used.

As stated, Coherence Therapy holds certain similarities with the DFI proposed in this manual. Firstly, in DFI the dilemmatic structure tends to remain unconscious to the patient; although when it is explored in session they usually recognize the dilemma as their own. Furthermore, Ecker and Hulley [51, 52] coincide with PCT in the importance of the sense of coherence in the patient’s life, and the pro-symptom position could be reflected in the congruent constructs forming an implicative dilemma. In both approaches, the symptom has a positive value in itself, not simply a secondary gain. DFI presents an important operational difference, though, in the way of identifying dilemmas, which is done with the RGT, and subsequently confirming these with the patient in the initial sessions. Another difference is that in Coherence Therapy the preferred step after the identification of the pro-symptom position (more recently termed “emotional implicit learning” or “symptom-requiring schema”) is its complete erasure from the emotional memory system or eradication [53]. For that purpose, they provoke a series of juxtaposition experiences in which the emotional learning which once made the symptom necessary is disconfirmed. This therapy process (fully described in [52, 53]) is paralleled by recent discoveries in neuroscience on the process of memory reconsolidation. In contrast to this focus of Coherence Therapy in juxtaposition and the consequential dissolution of the pro-symptom position, the DFI is aimed at assisting the patient in recognizing the dilemma and assisting him or her in finding a way to resolve it. Here, the therapist does not have a definite itinerary to dilemma resolution (like juxtaposition in Coherence Therapy) other than empowering patients to find their own way.

## Methods

### Specification of defining interventions

#### Unique and essential elements

The main ingredient that we can consider as unique for DFI is the identification of the dilemmas (as described in the section on symptoms/disorder assessment by the therapist in this manual) and also the focusing of therapy work on the elaboration of the dilemma(s) found. However, in addition to that, for the work with dilemmas a series of techniques and procedures are used and will be described in this and the following section.

##### Identification of dilemma’s prototypical figures

These figures can be found among the elements included in the RGT upon a visual inspection of their ratings. Prototypical figures are those significant others that fit the two positions depicted in the dilemma. For implicative dilemmas, on the one side (usually represented on the left, see Fig. 1) one or more elements are rated both on the congruent pole of the congruent construct and on the current pole of the discrepant construct (“generous” and “depressed” in the example of Fig. 1). On the other side (usually represented on the right), one or more elements are rated on the undesired pole of the congruent construct and on the desired pole of the discrepant construct (“selfish” and “happy” in the example). While the first set of elements represents the patient’s current position (and its implications) the latter set epitomizes the implications of change; that is, how is change envisioned as exemplified by other people. For dilemmatic constructs, prototypical figures would be those rated at either pole of those constructs using the more extreme scores within the range used by the patient.

Prototypical figures are used in the initial exploration of the dilemma with the patient (see session 1 below). They are used to visualize the terms of the dilemma in the context of the patient’s life. A formulation is suggested along the lines that according to his or her ratings in the grid there seem to be “two kinds of people” (in the example, those who are generous but depressed and those who are happy but selfish). Basing the presentation of the dilemma formulation in the specific ratings given by patients to these prototypical elements of their grid makes it more feasible for them to recognize the dilemma as their own and feel motivated to work towards its resolution.

##### The magic wand technique

This technique is used also in the initial phase in the early exploration of the dilemma in order to make it explicit in the conversation (see session 1). The general aim is to question the idea that change (in the discrepant construct) is completely desirable and to express some doubts about this change being completely positive for the patient. For that purpose, the therapist asks the patient if she or he would really be ready for change. This is done by inviting the patient to imagine a scenario in which the therapist can produce that change immediately with his or her magic wand. Even when the answer is “yes” the therapist questions the idea that change is so convenient for the patient. Previously identified prototypical figures are also used for that end. With this exploration some negative implications for change are made explicit along with the need to achieve it. In this way, the dilemmatic nature of change becomes fully visible in the eyes of both patient and therapist.

##### Self-characterization

Proposed by Kelly [4], this homework assignment is one of the forms of constructivist assessment [54] widely used among personal construct practitioners. This technique directly follows Kelly’s credulous approach according to which the views provided by the client are taken at face value as an expression of his or her experiential truth. As with the RGT, patients provide their own constructs but in this case this is done in a narrative form. For that, they have to write a self-description using the third person as if written by a cherished close friend. This writing assignment is prescribed to the patient at the end of the first session to be revised at the beginning of the second one.

##### Laddering up

This technique was introduced by Hinkle [55] with the aim of exploring the superordinate implications of a given construct. The underlying idea is that the meaning of a construct is better understood if the implications it has in terms of other constructs are known. In DFI the constructs for which this investigation is carried out are those involved in the dilemma. Stemming from the idea that each construct represents a pair of alternatives to choose from, laddering up permits exploring the superordinate implications of each one (ie, Why for this person it is better to be social than timid?). In carrying out this exploration patients reach a better understanding of their meaning system.

Laddering up is a semi-structured procedure used both in clinical and non-clinical contexts in which the interviewer selects a personal construct of the client and asks him or her to point to the desired pole of that construct and, then, to say why that pole is more preferable than the other. This is complemented with an inquiry about the implications of the undesired pole. This investigation leads to one or more constructs, for which the whole procedure is applied again, and so on until no more superordinate implications appear. The laddering up interview is carried out in the second session of DFI with the discrepant construct of the implicative dilemma (see session 2 below, for more details).

##### Laddering down

Also derived from the work of Hinkle [55], this procedure is aimed at revealing the subordinate implications of each of the poles of a given construct. It is particularly useful to avoid misunderstandings between therapists and clients. Indeed, because of the established meaning of words the therapist may take words uttered by their clients by their standard rather than their personal meaning. Laddering down is particularly used to explore verbal labels which seem too abstract, general (eg, “happy-unhappy”), vague or ambiguous. This is also a semi-structured interview in which the interviewer effectively asks for an operational definition of the personal construct. Some examples of questions for that investigation are: What kind of person is a “happy” person? How can you know that someone is happy? What are the characteristics of someone who is happy? Laddering down is applied in DFI, if necessary, with the discrepant construct of the implicative dilemma or with a dilemmatic construct, in the second session.

##### Dialectical laddering

Based on the laddering up procedure devised by Hinkle [55], Neimeyer [54] proposed a variation that fits very well into the work with dilemmas. Dialectical laddering is particularly appropriate whenever the patient is unable to identify a clear value preference between the two poles of a construct (a situation overtly signaling a dilemma), which impedes any further laddering up. In DFI this is precisely the case for dilemmatic constructs. The aim here is to reconcile these poles in a higher order integration or synthesis. For that goal, the therapist assists the patient in finding an integrative alternative to the initial construct. Thus, the main function of dialectical laddering here is not only the exploration of the implications of the construct under investigation but also (and mainly) to make one step in the change process by creating alternative meanings. Once the new label has been elicited the therapist asks for its opposite pole so that a new, more integrative construct is created. For example, for the dilemmatic construct “gives everything-keeps everything for oneself”, the person might provide an alternative label, “extremist,” encompassing both poles, for which the opposite pole could be “moderate”. In this new construct the patient might have no problem in voicing his or her preference for “moderate”. This way the dilemmatic construct (one without a preferred pole) would have evolved into a new construct with a clear preference.

#### Essential but not unique elements

##### Therapeutic relationship

In PCT psychotherapy cannot be equated with the application of a series of techniques deemed appropriate for a given problem or diagnosis. Rather, it is seen as a conjoint exploration of the, often conflictual, meanings of the symptom or problem in the context of the client’s life. Therefore, the view of the therapist as “the expert” who leads the therapy process by prescribing the “right” techniques, a conception inherited from the medical model, is not embraced in constructivist approaches (including DFI). In PCT the therapeutic relationship is understood as a cooperative endeavor between two experts with different kinds of expertise [56]. Clients are experts in the contents (themes, goals, projects, experiences) of their own lives and therapists are experts in the processes of construction and their two-way influence in emotions and actions, in the dynamics of change, in the way relationships develop and, in particular, in the therapy as process and context. In this cooperative venture it becomes essential to distinguish between the two domains of expertise of the issues dealt with in the therapy process and also to respect the issues that lie within the client’s domain.

This constructivist view of the therapeutic relationship may partially overlap with cognitive therapy’s assumption of “collaborative empiricism” [34] and, in different ways, with some experiential and phenomenological approaches. Maybe some of its more unique characteristics can become more evident in combination with the aspects which follow.

##### Communication style of the therapist and the invitational mood

Constructivist therapists’ communication style is seldom directive or prescriptive; rather, it reflects curiosity in the client’s world and personal, often idiosyncratic, meanings. Their style is reflective and invitational, that is, based in Kelly’s “invitational mood” [4]. He invited his clients to consider the implications for different ways of construing a given event. Thus, using this invitational mood, a therapist may invite the client to entertain seemingly contradictory constructions of the same thing in order to explore where each construction leads. In this way, the therapist does not remain wedded to a particular construction (even when that construction has been suggested by him or her) and along with his or her client they can shift among various ways of construing the same circumstances for the sake of exploration. They do not need to consider those constructions as either true or false (nor as shrewd revelation about a previously hidden issue) to explore their implications for the meanings and life of the client. An example of this kind of attitude in DFI could be “Imagine that it would be possible for you to be happy without becoming a selfish person, how would that be?”

The exploration of hypothetical situations, also called feed-forward questions, became a modality of circular questioning in the systemic model about 30 years ago [57, 58]. Also, the invitational mood can be seen as similar to a certain way of questioning automatic thoughts and beliefs in cognitive therapy [59].

##### Tschudi’s ABC

This technique, proposed by Tschudi [48] (see [60] for an update), is aimed at the exploration of positive and negative implications (advantages and disadvantages) of change, and also of not changing. As said before, it resembles the decisional balance sheet used in motivational interviewing, but the theoretical base of this technique is Kelly’s choice corollary, which underlines the capacity of human beings for making choices (something not so evident in many psychological theories) [4]. This corollary asserts that choices are elaborative, that is, an alternative is chosen if it offers the best prospect for anticipating events. Also in the corollary, Kelly specifies that elaboration can follow two different directions: extension or greater definition of the construct system, or in other words novelty or safety. This theoretical formulation is openly compatible with the possibility of some situations involving constructs in which each of the poles follow the different directions mentioned above, thus creating a conflict or dilemma which deserves exploration.

The procedure begins by taking the discrepant construct (here termed “A”), which includes a problem position at one pole and a desired position at the other, and asking the patient for the disadvantages of the former (eg, “depressed”) and the advantages of the latter (eg, “happy”). In their answer to this question, patients provide “B” constructs, that is, those that show the positive implications of change and the negative implications of remaining in a problematic position. These constructs represent the reasons for change and furnish the motivation for change. The final step consists of asking patients for the advantages of remaining in the problematic position of the “A” construct and also for the disadvantages of the desired position. In this way, when “C” constructs are provided by patients, less evident reasons for not changing are made explicit and help to understand their difficulties or “resistance” to change.

##### Reconstruction of immediate experiencing as a function of the dilemma

**Fig. 2:**
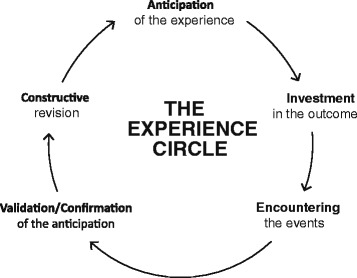
The Experience Cycle

This technique is derived from what Kelly termed “controlled elaboration” [4], a process in which therapist and client examine the constructions at play in a given situation moment by moment and how they shaped the client’s experience. Reconstructing the experience of the client is guided by the experience cycle of PCT [6, 9, 10], which describes the process of living as a continuous “experiment” in which our constructions are tested out and get eventually revised. One phase of the cycle refers to the **anticipations** we place on every one of our experiences, that is, the meanings involved in the experience under analysis. Most of those anticipations about the upcoming event are not conscious or explicit but can become so with this work. The subsequent phase of the experience cycle, **investment**, refers to the portion of core role structure involved in the construction of the event. It can be grasped with a question: To what extent is this situation or event important to me? The following phase refers to the **encounter** with the event, and it can be revealed with simple questions such as “What happened exactly?” The next phase involves an evaluation of whether what happened was a **confirmation or disconfirmation** of the anticipation at stake. Kelly remarked the role of personal construction also in this phase when he asserted that “validation represents the compatibility (subjectively construed) between one's prediction and the outcome he observes” [4; p. 158]. According to this perceived outcome, **constructive revision** of the construct system can occur, an essential process for human development and change (see Fig. 2 for a representation of this cycle). The experience cycle is conceived as a continuous process evolving moment by moment in our current life. According to Kelly [61], this cycle is the base for optimal functioning, that is, a process of inquiry and experimentation concerning the possibilities of the self. However, this process is constrained by a construct system which is often too limited. Also, it can be blocked at any of the phases of the cycle [62], which can lead to a variety of problems and symptoms that patients bring to therapy. For that reason, revising the construing process involved in relevant experiences can be useful in identifying the personal meanings involved and also in detecting both limitations and potential alternatives. As previously suggested [1, 63], the process of revising the construction of the client’s experiences is a common fundamental process of most psychotherapies.

In the third session of DFI the reconstruction of immediate experiences is carried out in the light of the dilemma which is being explored. Thus, therapist and patient work together to revise the phases of the experience cycle by investigating which were the anticipations involved in problematic experiences relevant for the dilemma, how central these anticipations were for the patient’s sense of identity, and how the event actually occurred. Then, the significance of the event is considered in the light of whether the anticipations involved were validated or not, and how that outcome required revising the construct system (maybe resolving the dilemmatic implications) from which new anticipations will be derived. In this process, the patient is asked to describe in full detail all the sensations, thoughts, actions and emotions occurring both in the revised experience and also in the process of revising it with the therapist. In this way, the events are explored in the light of the meanings involved and, especially, of the constructs forming the dilemma.

##### Analysis of the relational implications of the dilemma: Are there accomplices of the dilemma?

As mentioned above, PCT has a strong relational emphasis because it grants a central role to interpersonal relationships in the origin of personal meanings. In this sense it is similar to other approaches, in particular to interpersonal [64] and systemic therapies (although in DFI family members are not invited to the sessions). The aim in DFI in this respect is to analyse the role of significant others (usually family members) in the creation and maintenance of the dilemma which is being investigated. The goal here is to facilitate awareness in both patient and therapist of how these people influence the patient’s constructions of reality as a way to stimulate alternative constructions and roles less dependent on those of these figures. In this kind of analysis sometimes the notion of “accomplices of the dilemma” may be invoked to refer to the people who fully represent one or other sides of the dilemma (this work would be similar to the Identification of the dilemma’s prototypical figures described above), or also to those people who expect the patient to act in a way which corresponds to the side of the dilemma in which corresponds to the patient’s current functioning. These significant others might not welcome the client’s desired change nor the implications it has for the relationship. These (usually) family members are called “accomplices” of the dilemma because they treat the patient in a way which validates his or her current position in the dilemma (desired pole of the congruent construct and symptom pole of the discrepant one) and provide invalidation when faced with the patient’s attempts for change in his or her position or role in the relationship. Even when these family members welcome the change in the discrepant construct, they react negatively with respect to the congruent construct. For example, one or more family members might be glad to see the patient happy but complain that recently the patient is more “selfish”. In this way, the association between “happy” and “selfish” (the dilemma) is being implicitly confirmed in the context of one or more patient’s significant relationships.

In order to explore and, eventually, identify the accomplices of the dilemma the patient is asked to describe problematic situations with significant others involving the dilemma being explored. Particular attention is paid to the patient’s constructions of these significant others in terms of the constructs involved in the dilemma: How are they according to those constructs? What do they expect from the patient in that situation? How would they react if the patient doesn’t meet their expectations? Particular attention is paid to the people who might be impeding change, overtly or covertly, and to the behaviors or comments they make in that respect. The patient is, then, asked to search for alternative ways of handling that situation.

A second part of this work consists of thinking of significant others (maybe some elements in their grid) who can be described using the desired poles of both congruent and discrepant constructs. These significant others would be regarded as exceptions to the dilemma (in the example, they are both “happy” and “generous”). Once identified, the patient is asked to consider how these people would act if they would be involved in the problematic situation which has been previously analyzed. These can help the patient in finding other ways to manage this kind of problematic situation; ways which will hopefully reflect a resolution of the dilemma (see content of session 4).

##### Historical reconstruction of the dilemma

Life review is an ingredient of a variety of psychotherapy schools, ranging from psychoanalysis to schema focused therapy [65]. In DFI the investigation of the life history of the patient is not aimed to identify a specific situation in the history of the patient which would be considered the origin of the dilemma. The intention is not to identify the guilty one(s) nor to focus the therapy in the past but, rather, to try to find the reasons for the patient’s present constructions [66] precisely to lessen any feeling of guilt or shame with respect to having the symptom(s). In other words, the goal of the historical reconstruction of the dilemma is to reach a conjoint understanding of how the dilemma was “logical” in a given historical context of the life of the patient, a therapeutic maneuver that Kelly termed “time binding” [4]. This kind of reconstruction facilitates the search for alternative constructions more suitable for the present time. Also, this approach favors the generation of new constructions which respect and are compatible with the need for continuity in the sense of identity across the course of a person’s life. In sum, with the historical reconstruction of the dilemma an extensive understanding can be achieved of how the dilemma was formed as a way to make sense of a given situation, and how it has been active and relevant across the patient’s life, including the present time and perspectives to a future without the dilemma.

In DFI the historical reconstruction of the dilemma is prompted by a task assignment titled “chapters of the autobiography”. It consists of asking patients to write the title of the set of chapters that would form their autobiography, and the time span each one covers, as if they would have to write it. Patients have to decide on the number of chapters and their time coverage as a function of the landmarks they consider meaningful in their lives (even when these chapters have nothing to do with the dilemma being explored). Patients are also asked to provide a title for a chapter about the future, a chapter describing their life with the dilemma solved. This list of chapters is then discussed in the therapy room, and patients are asked to identify their position in the dilemma in each of the chapters. They are also asked to recall episodes in their lives in which the dilemma was relevant, and also to examine their position in the dilemma across their life span up to now. The future chapter is also discussed paying attention to its consistency with respect to previous chapters. The change that is wanted for the future cannot be envisioned as perfect or ideal but as an evolution of the self which is coherent with the patient’s personal history (see content of session 5).

Also in the context of the historical work with the dilemma, patients are asked to write the “history of the dilemma,” which should include the origin of the dilemma, an account of its influence across their lives, its importance in the present moment, and possible ways of resolving it. This task assignment is based on the recognition of narrative as an organizing principle of human experience (e.g., [67]) and on the extensive evidence on the benefits of therapeutic writing for the physical and mental health of individuals [68]. One explanation of these positive effects is that writing about one’s problematic experiences helps people to reorganize thoughts and feelings, and also to produce more coherent and meaningful narratives about the events of their lives [69].

##### Dramatic representation of the dilemma

Influenced by Jacob Levy Moreno, the creator of psychodrama, Kelly was a pioneer in the use of enactment in psychotherapy (his fixed-role therapy can be seen as an example) [4]. DFI employs the two-chair dialog technique from Gestalt Therapy (also heavily influenced by Moreno). Gestalt and experiential therapists use this technique with the purpose of integrating parts of the self in conflict. For that they use two chairs each representing one of these parts, and ask the client to express one of the parts while sitting in one chair and the other expressed while sitting in the other one, switching from one chair to the other as a way to stimulate a dialogue between the parts aimed at their integration. In DFI the dialogue is between the two parts of the dilemma, also with the goal of their integration. Thus, one chair represents the present pole of the discrepant construct, which is connected to the congruent pole of the congruent construct, and the other chair represents the desired pole of the discrepant construct, which is linked to the undesired pole of the congruent construct. The therapist acts as a facilitator of this dialog with the goal of reaching an integration (or dilemma resolution) which takes into account the needs expressed in each chair.

##### Future projection: life without the dilemma

Often the dilemma being explored has been present in the life of patients for many years. Thus, they have felt blocked for a long time, not finding a proper solution for the dilemma, which makes them feel depressed, isolated or passive. In these cases, just imagining how life could be without the dilemma might be quite difficult. In other cases, the dilemma may have emerged at a given point in their life in a way that required a change in the view of self, this leading to guilt and/or shame or even confusion (“I was not like that before”). In all of these cases it is important to elaborate a future perspective which is consistent with their personal history while including more viable alternative constructions.

Milton Erickson [70] created a procedure in which he asked his clients under trance to project themselves into a time in the future in which their problems will be solved. Then, he conversed with them about how they had managed to solve their problems. Following this inspiration, De Shazer [71] developed the “crystal ball technique” and the “miracle question”, a keystone in Solution-Focused Brief Therapy. This influence was also extended to other systemic authors [58]. In DFI, a similar approach is followed. Patients are asked to imagine how life would be without the dilemma, and to describe specific instances showing that it is solved. For this purpose, a variant of the “magic wand” technique (used in the first session) is employed but with a focus on the future. This time, the therapist asks patients to imagine in detail how life will be once the dilemma is solved (see session 7). Particular attention is paid to the relational implications of dilemma resolution and also to the coherence of this new achieved personal identity. The goal here is to picture a scene as detailed and precise as possible of how life would be without the dilemma. For this, the therapist asks patients to give a more detailed description (e.g., “I will wake up at 8 a.m. and then ….”) whenever more general descriptions are provided (“I will be more active”).

Unlike the miracle question usually employed at the beginning of the therapy process in the solution-focused approach, the magic wand technique here is used towards the end of the DFI to construct an image of the future life of the patient which incorporates the contents elaborated across the previous therapy sessions. This picture of the future life of the patient should integrate both sides of the dilemma as represented by the discrepant and the congruent construct. In DFI this kind of therapeutic activity aimed to promote change in a direct way is left to the end of the process to avoid an excessively positive view of change that would conceal the dilemmatic nature of change, the reasons why the change has not already occurred spontaneously. Once the negative implications of change have been fully explored the change depicted for the future can be more coherent with the patient’s sense of identity. For that reason, the change that is sought is one that is compatible with the continuity of the self of the patient, not an ideal or abstract one, not a change that involves a rupture of that continuity.

##### Searching for alternatives to the dilemma

Stemming with the image for the future created in the projection procedure described above, patient and therapist work together to find alternatives to the dilemma (see contents of session 7). First, specific instances or episodes reflecting a resolution of the dilemma already happening in the present life of the patients are searched for. In these moments patients have acted *as if* the dilemma were solved. Their feelings and sensations are explored in detail, paying attention also to significant others’ reactions. If a patient is not able to find any of those instances this kind of exploration is done with respect to any past situation of that kind. We know that exceptions can be found to problems even in lives with a problem-saturated narrative. Likewise, exceptions to the dilemma can be found as well. In this respect, DFI parallels narrative (e.g., [72]) and solution-focused (e.g., [71]) therapies although the goal here is the search for alternative constructions to the dilemma more than to the problem.

The search for alternative constructions is also an ingredient in the cognitive-behavioral therapies but in PCT it has different emphasis. As stated by Kelly [73] “… to entertain some novel hypotheses about other ways of living, he can save himself and his therapist a lot of trouble overcoming the ‘resistances’ and ‘false premises’ of his previous outlook. Therapy could then become concerned with alternatives instead of involving the participants in long, intricate, and reductionistic analyses designed to disabuse the client of his ‘neurotic’ notions” (p. 55). Interestingly, we could equate the “neurotic notions” alluded by Kelly here in this quote with both distorted thoughts/irrational beliefs and faulty defense mechanisms. Effectively, in this assertion Kelly contrasts PCT’s focus on construction of alternatives from those approaches which focus their work on analyzing what the patient is doing wrong and trying to correct it.

#### Recommended elements

Therapists using DFI are encouraged to fully respect, rather than challenge, the patients’ constructions, taking into consideration the awareness that they were useful for making sense of reality in the past. This respect is also based in the recognition of the need for continuity in the sense of self-identity, even more important than the need for change. For this, careful attention is paid to the identification of patients’ core constructs and to the need of preservation of the congruent pole of the congruent construct of the dilemma. The attitude required for this approach requires a decentering of the therapist’s own constructions by taking into account the patients’ constructions of their problems, self and significant others.

#### Proscribed elements

Based on the conception of the therapeutic relationship assumed in PCT, the techniques being applied and the task assignments are always agreed upon with the patient and never directly nor indirectly imposed. Rather than being directive or prescriptive, therapists should adopt a curious and “invitational” stance. In PCT techniques are conceived as forms of exploration of new meanings, and therapists’ comments are presented to the client as suggestions or alternative possible constructions and should not be expressed as statements about the external or internal reality of the patient.

Similarly, the particular solution found for the dilemma, whether it involves a change in the patients’ position on the discrepant construct or not, cannot be decided by the therapist but rather by the patients themselves. In DFI, instead of pushing for a pre-established change therapists promote the exploration of alternatives. In fact, a recent study [74] comparing good and poor outcome cases treated with DFI found that dilemmas were resolved mostly using two pathways to change: (1) reducing self-ideal discrepancy; and (2) lessening the strength of the association between the congruent and the discrepant construct.

### General format

#### Format for delivery

This manual presents eight individual therapy sessions to be carried out after seven group sessions (received previously following a cognitive-behavioral group therapy format, although it would also be compatible with other kinds of intervention). After the individual treatment module a final group session is conducted to bring the therapeutic process to an end.

#### Frequency and intensity of sessions

The sessions are 1 h long and are carried out weekly.

#### Flexibility in content

Although detailed session to-session protocols seem to leave little room for flexibility matching the patients’ expectations and needs is a central challenge of therapy manuals and, as it can be deduced from the focus on the patients’ personal meanings described above, it is essential for DFI as well. Thus, feedback on the feelings and impressions experienced by patients in relation to the techniques or procedures employed as well about their symptom progress (using instruments such as CORE-OM [31]) will be discussed conjointly with the therapist, who will have to adapt the manual implementation to each patient.

One way to allow for flexibility in the manual is in the sessions’ structure. Seven of the sessions have specific content which covers different aspects related to the dilemma’s elaboration (as specified below). But there is also a “wildcard” session dedicated to further the work done in one of the sessions which, according to the therapist, may require additional time. There is also the possibility that the “wildcard” session ultimately not be used, thus maintaining the seven sessions of treatment. In the section explaining session content detailed information is provided to help the therapist decide on the use of this “wildcard” session.

Although detailed session to-session protocols seem to leave little room for flexibility matching the patients’ expectations and needs is a central challenge of therapy manuals and, as it can be deduced from the focus on the patients’ personal meanings described above, it is essential for DFI as well. Thus, feedback on the feelings and impressions experienced by patients in relation to the techniques or procedures employed as well about their symptom progress (using instruments such as CORE-OM [31]) will be discussed conjointly with the therapist, who will have to adapt the manual implementation to each patient.

#### Session format and structure

Each session has content that is presented and justified to the patient. The therapist explores whether the patient agrees to work on the content, asks permission to do so, and negotiates alternatives (as close to the planned content as possible) when necessary.

The sessions begin with the review of homework assignments (if any). Once this is done, we ask for any aspect (situation, thought,…) which has occurred during the week and which may be relevant to the dilemma’s elaboration. Next, the session content is presented to the patient and, if there is agreement, the corresponding activities are carried out. If there is no such agreement, the patient’s reluctance or difficulties are attended to and a negotiation of the activities to be carried out in session is attempted, trying to adapt them as much as possible to the content planned for the present session or any other of the sessions contained in the program. Before the end of the session the therapist enquires about the session’s content, ie, what aspects the patient found more relevant, useful or interesting. Finally, a task may be assigned for homework, which will be addressed in the next session.

The session content follows an inclusive logic, in such a way that the content for each session may include everything previously worked on in earlier sessions. The therapist must always keep in mind those aspects discussed in previous sessions so as to relate them to the new content which emerges in conversation, in such a way that the themes are spun in a narrative that becomes increasingly more structured and consistent with the dilemma (or dilemmas) being worked on. For example, in session five, dedicated to the historical reconstruction of the dilemma, the therapist must keep in mind the relational aspects addressed in the previous session, in order to explore how these have been modified throughout the patient’s history.

#### Extra-session tasks

Some of the sessions are supported with homework assignments, generally of a narrative type. The session content includes detailed information on these activities. The purpose of the tasks is twofold. On the one hand, they help in the elaboration of the dilemma per se, and on the other hand, they offer material to work on in the sessions.

#### Required training for the DFI therapist

As said at the beginning of the introductory section, DFI can be considered as an adjunct to other more comprehensive therapies even to those which are not based in constructivist epistemology. However, for the therapist applying this part of the treatment -the DFI – a sound training on constructivist psychotherapy [6–8] is needed. This training should not only consist of the foundations for understanding human functioning and the process of symptom formation but also practice-based learning. Communicative style of the therapist, case formulation and the mastery of the set of techniques included in DFI are key aspects of a proper training for these therapists.

Previous training and experience of the therapists will facilitate the learning of the array of techniques included in DFI. For example, those with a CBT background might feel more comfortable with techniques such as laddering and Tschudi’s ABC because these procedures involve a systematic exploration of cognitions, and those who undergone a Gestalt or emotion focused therapy training will exhibit more competence in the two-chair dialogue. Anyhow, what is essential for DFI is to apply the techniques adapted to the specific goal of dilemma exploration and resolution. Following with the example above, a therapist with CBT training will have to bear in mind that, despite its apparent similarity with cognitive restructuring laddering is not aimed at discovering cognitive distortions or thinking errors but to explore the personal construct system to grasp a picture of the implicit and explicit meaning the client is ascribing to symptoms.

On the other hand, competence in the administration and analysis of the RGT is also capital since this a crucial ingredient of the assessment process and case formulation. Training in the RGT cannot consist only of reading manuals and following their instructions. Rather, supervised practice with repertory grid administration (to a few volunteer interviewees) and data analysis are strongly recommended before using it with a proper client. Currently, the Master on Cognitive Social Therapy (see https://es.scribd.com/doc/235672680/La-Terapia-Cognitivo-Social-un-Enfoque-Constructivista) of the *Universitat de Barcelona* provides a comprehensive training program in constructivist psychotherapy with a specific focus in dilemma work. Advanced students in this program participated as therapists in the controlled study [2] testing the efficacy of DFI (now in follow-up data collection phase) but even with these therapists an intensive training based on the present manual was performed. It consisted in workshops (4 to 8 h in total for these therapists with good training in constructivist psychotherapy) using role playing and detailed materials from clinical cases. In a small group context, these workshops allow therapists to experience both roles (patient and therapist) and to receive feedback from supervisors and other group members. While they conduct their own cases in the real life context participation in a supervision group using their audio tapes of the sessions is required to complement their training in DFI.

### Session contents

#### Session 1: dilemma presentation

The aim of the first session is to present the dilemma, explore its relation to the patient’s demand and involve the patient in the dilemma-focused intervention. Being the first session of the individual treatment module, it is necessary to address aspects related to the therapeutic relationship and the format of therapy, before focusing the session on the dilemma(s) presentation. Specifically:

- Assess how the client is feeling, the changes that may have occurred since the end of the group therapy and the factors involved. This aspect may be especially important when the questionnaire scores have worsened or an increased risk of suicide is detected.
- One of the important objectives of this session is to begin to establish a good therapeutic alliance. In this sense it is important to bear in mind the concepts of active listening, empathy, authenticity or acceptance and communication skills.
- Description of the new “therapy framework” (group vs. individual). Part of the first session is spent exploring how the person has found the group sessions, without focusing on comments about other participants unless they are relevant to the dilemmas found during the assessment. The therapist explains the differences of this new therapy format in more or less these words: “These sessions we are going to do individually are somewhat different to the ones you have already done with the group. In this case we will focus exclusively on the things that are relevant to you, those that worry you or that which you would like to change. We will also be working from one of the instruments you used during the assessment phase, the grid technique, which we will discuss in today’s session.”
- Revision of the patient’s expectations in relation to therapy, after the group stage, once part of the treatment has already been completed.

The bulk of the session consists in the presentation of the dilemmas to the patient based on the exploration of prototypical figures. To this aim a series of steps are followed:

1. Presentation of the discrepant constructs implicated in the dilemmas and/or dilemmatic constructs.
  1.1 Example with Dilemma(s)’ Discrepant construct(s)
    Therapist: According to the scores you gave in this questionnaire, it seems you feel quite depressed at the moment, but you would like to be happy, is that so?
    Patient: Yes, yes, I am quite depressed now.
    T: I also see that you consider yourself a very private person and you would like to be very sociable, is that so?
    P: Yes.
  1.2 Example with Dilemmatic Construct(s)
    T: From what I have seen in the scores you gave in this questionnaire it seems that for you, between being a passive or an aggressive person, your ideal is a 4, that is to say, a midpoint. What does that mean to you?
    P: Well I don’t know… that I think it’s best not to be passive nor aggressive either…
2. Selection of the discrepant/dilemmatic construct to work on (if there is more than one). To this aim we enquire on the importance and relation they have to the patient’s current problem.
  T: All these are things that we can work on in the following sessions, would it be ok with you if we dedicated the sessions to one of these aspects you would like to change?
  P: Yes
  T: Which do you think is the most important to you? Which would you like to address first?
3. Exploration of the dilemma focusing on prototypical figures. Below are some examples of questions that can guide this exploration. There is no specific order in which to ask the questions; rather, the aim is to start a dialogue in which the two parts of the dilemma begin to appear in the conversation.
  3.1 Example questions for exploring dilemma(s)’ discrepant construct(s):
    How would you describe depressed people in general?
    How would you describe happy people?
    From what you say, your friend Pere (element in the grid) is a very happy person, as you would like to be. He is also quite active and cheerful… but maybe there are other aspects of Pere that are not so nice.
    (Allow space for if the patient offers one of these negative aspects)
    According to your point of view Pere is quite selfish and cares only about himself. Is that so? (explore also other examples)
  3.2 Example questions for exploring dilemmatic construct(s):
    How would you say aggressive people are, in general?
    And passive ones?
    From what you say, your friend Elena (element in the grid) is very aggressive, while your mother (element in the grid) is quite passive. It seems you would not like to resemble any of them, as in this trait they are not how you would like to be. Is that so?
4. Presentation of the dilemma as a work goal in therapy.
  4.1 Example with implicative dilemma
    T: Although Pere is very happy, it seems he is not the type of person you would like to be… Imagine if I were to have a magic wand (show a pencil) and I could quickly turn you into a happy person, like Pere, would you want me to do so?
    P: Maybe not…
    T: Why would that be?
    P: Often happy people don’t preoccupy themselves with people who aren’t happy, they don’t care much about others because they are already happy…
    T: It is as if being sad or depressed helps you bear in mind other people’s feelings… Would it seem useful to you if we dedicated these sessions to explore how you can be happy without disregarding other people’s feelings?
  4.2 Example with a dilemmatic construct
    T: Sometimes it is complicated to stay in a middle point… You need a lot of balance! It is as if one finds oneself facing a dilemma with each situation: Should I be more aggressive? Less aggressive? More passive? Less? Sometimes one can feel blocked when facing so many decisions. Have you ever felt this way? Would it seem useful if we dedicated these sessions to finding a good solution or a new perspective for this dilemma?

Although dilemmas include constructs that show a discrepancy between how the patient sees him or herself now and how he or she would like to be (ideal self), on occasions the relationship between the patient’s demand and the dilemma’s resolution is not obvious and it becomes necessary to explore and elaborate the possible relationship. It is important to make a connection, if possible, between the dilemma and the patient’s explicit demand, thus ensuring, insofar as possible, that the work with the dilemma will result in the patient’s desired changes. This connection could be made with these or similar words: “You were saying that what you would like to achieve the most with this therapy is [GOAL. For example, “improve my mood”], do you think that what we have been talking about today [DILEMMA. For example, “to achieve the goal of being more sociable without giving up being friendly, warm”] has to do with [GOAL. For example, your mood]? Do you think that if we resolve this dilemma your mood might also be benefited?”

At the end of the session we ask for a **self-characterization** as an extra-session task, explaining that it helps to better know him or her and to continue exploring the dilemma that we have been talking about in session. The instructions for the self-characterization are as follows: I would like to ask you to do a task at home that we will work on in the next session, and that will help us to further explore this dilemma that we have been talking about today. I would like you to write a brief characterization of (patient’s name) as if he (or she) were the character in a theatre play. Write it as a good friend who knows you intimately and very sympathetically, better even than maybe anyone else could know you. Make sure to write it in the third person. For example, start by saying “(patient’s name) is…”

#### Session 1: troubleshooting

1. The patient has difficulty selecting a discrepant/dilemmatic construct to work on. The therapist must explore these difficulties, reassuring the patient about this decision: one may begin working on an aspect, but, if the patient considers it necessary, it is possible to work on another construct during the sessions. On other occasions, various discrepant constructs make up a single implicative dilemma. In these cases it would not be necessary for the patient to choose a single construct to work on.
2. The discrepant/dilemmatic construct seems of little relevance to the patient’s problem. For example, a dilemma is identified with the discrepant construct “punctual-tardy” (congruent construct: “spontaneous-rigid”). It becomes necessary to explore, at least partially, during the first session, the superordinate implications of the construct punctual-tardy, to understand the hierarchical net which constitutes its framework. Thus, when seeing its relevance in the construct system, the patient can select it as a construct to work on. The therapist may explain that all these traits are “connected” and that sometimes seemingly superficial changes may represent bigger changes.

#### Session 2: dilemma elaboration

The aim of the second session is to continue working with the dilemma, searching for the nuances, the details, etc. that are relevant to the person, to achieve a more comprehensive understanding of the dilemma’s implications. The first part of the session is dedicated to the revision of the homework assignment, the self-characterization, with special attention to the elements of the dilemma that appear in the writing. In this description the following aspects are explored:

- *Empathic reading*: how does one see the world through these eyes?
- *The first part of the writing:* usually constitutes a “safe base” on which to elaborate other aspects, the presentation the person uses when talking about him or herself. In this presentation we can often find the congruent constructs that are part of the dilemma and tend to be more nuclear in the sense of oneself.
- *Repeated terms or with similar themes:* indicate a greater weight in the person’s construct system. The therapist should pay special attention to those which reflect both poles of the dilemma, not only in the same terms used in the repertory grid, but also all those synonyms, paraphrases, expressions, etc. that lead us back to the dilemma, given that the narrative nature of this technique allows for a greater freedom of expression. If such phrasing is more appropriate for the patient, it is used hereafter to refer to the dilemma.
- *Causal analysis*: Do we encounter causal explanations of this dilemma? For example, “a betrayal changed me (implied: before I was different, naïve)”, “my mother was the same as me (implied: it is genetic, inheritance)”, “as my father never loved me (implied: I will not be able to change or do any good)”. It is important to pay attention to these causal theories so as to address them in so far as they affect the change process itself.

**Fig. 3:**
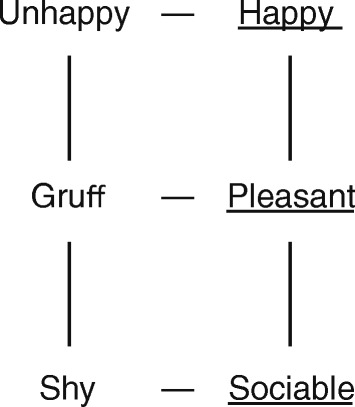
Example of the Laddering up technique

**Fig. 4:**
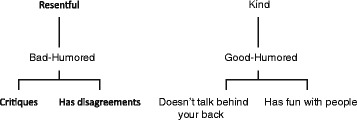
Example of the Laddering down technique

**Fig. 5:**
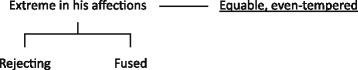
Example of the Dialectical laddering technique

In order to explore the implications of the dilemma, one of the following techniques is selected:

- *Laddering up*. This technique is appropriate in those cases where we are working with an implicative dilemma, in which case we use laddering up with the discrepant pole. It is not suitable to use laddering in dilemmatic construct cases, as the intrinsic difficulties selecting the desired pole would be an impediment to initiate this laddering procedure. It would not be appropriate either to use laddering up when the discrepant construct already presents a high level of abstraction (eg “happy-unhappy”). For example, with the discrepant construct “shy” vs. “sociable” (desired pole) the questions could be: “Why is being sociable preferable to you?” or, alternatively, “Why is being shy not desirable to you?”.
  The new construct that emerges from the reply to these questions constitutes a new rung in the ladder, where we ask for the preferred pole (when writing it down we underline it) and the reasons for its preference. The process is repeated until the patient cannot explain why they prefer a certain pole or when all the replies given are similar (see Fig. 3).
- *Laddering down*. This technique is appropriate when the dilemma’s labels have a high level of abstraction and our aim is to know what concrete meaning the person gives to the term. It is also appropriate to use with dilemmatic constructs, to understand why both poles are considered positive or negative. It would not be appropriate to use laddering down when the constructs are very specific (eg, “punctual-unpunctual”). For example with the discrepant construct “resentful-kind”, the questions could be: “How can you see when someone is kind?”
  “How would you tell if someone is kind?”
  “How do people know if one is resentful?”
  For each reply we enquire about the opposite. The process is repeated until the person is unable to offer new answers. On one of the rungs the patient may give more than one answer, focusing the following analysis on the one considered more relevant to the person’s current problem (see Fig. 4).
- *Dialectical laddering*. This technique is especially appropriate when working with a dilemmatic construct. For example, with “rejecting-fused” the questions could be:
  “If you had to find a label that encompassed both of these characteristics at the same time, to integrate them in some way, what would this label be?”
  “Can you think what these two characteristics might have in common?”
  After achieving this synthesis, we ask what the opposite would be. With this new construct, we ask the patient to indicate which would be his or her preferred pole. If they are unable to choose one of them, the process is repeated (see Fig. 5).
- *Tschudi’s ABC*. This technique is appropriate to explore both the discrepant construct in the implicative dilemma and the dilemmatic construct (see Table 1).

Finally, at the end of the session, it is necessary to strengthen the relationship between the dilemmas and the patient’s complaint(s). It is advisable that this relationship be highlighted during the first sessions, in order to ensure that the patient establishes the said connection and perceives that the change is headed in the desired direction. For example, for an implicative dilemma including “humble-arrogant” as congruent construct and “shy-sociable” as the discrepant one, if the patient’s demand is “to be happier and to improve my mood” we suggest saying something like the comment as follows.

“As we were saying in the previous session, it seems that resolving this dilemma would help you to improve your mood. If you succeed in becoming a more sociable person and still be friendly and kind with others, you would feel happier. After what we have seen in today’s session, what do you think about this? In which sense would you be happier?”

#### Session 2: troubleshooting

The therapist acts in an excessively “technical” way when applying the procedure, losing sight of the session’s objective. The therapist feels “without freedom” to establish a productive dialogue with the patient. It is essential to remember that all these techniques are just “means” to get to know the patient’s constructions; they are not “ends” or goals in themselves. It is recommended to maintain an attitude of curiosity towards what the patient explains and to be attentive to the questions that suggest the patient’s explanations.

#### Session 3: reconstruction of immediate experience

The goal of this session is to nail down the notion of dilemma, its manifestation in the patient’s daily life. To this aim we proceed to the reconstruction of immediate experience based on the dilemma, following the subsequent steps:

- A recent episode in which the dilemma was involved is selected and the patient is asked to describe it in detail from start to finish.
- Throughout the whole description, the therapist asks for details about how the patient felt in that moment, what he or she was thinking, what he or she did, etc.
- We explore in particular detail the moments where emotions appear either explicitly or implicitly (in this case, make them explicit) to make a connection with the meanings attributed by the patient to the situation.
- We explore the experience of the dilemma in this situation: how the congruent construct manifests itself, qualities of this experience and emotions that appear, etc. Following the example shown in the graph, the objective is to explore if somewhat positive emotions exist in the narrated episode, when feeling “humble” in front of others who have shown “arrogance”.
- All of this is aimed towards obtaining the implied meanings in the construction of the experience, the anticipations, often barely conscious, which guide their actions, and which become invalidated with the experience, with consequent negative emotions (or positive emotions in the case of validation).

**Fig. 6:**
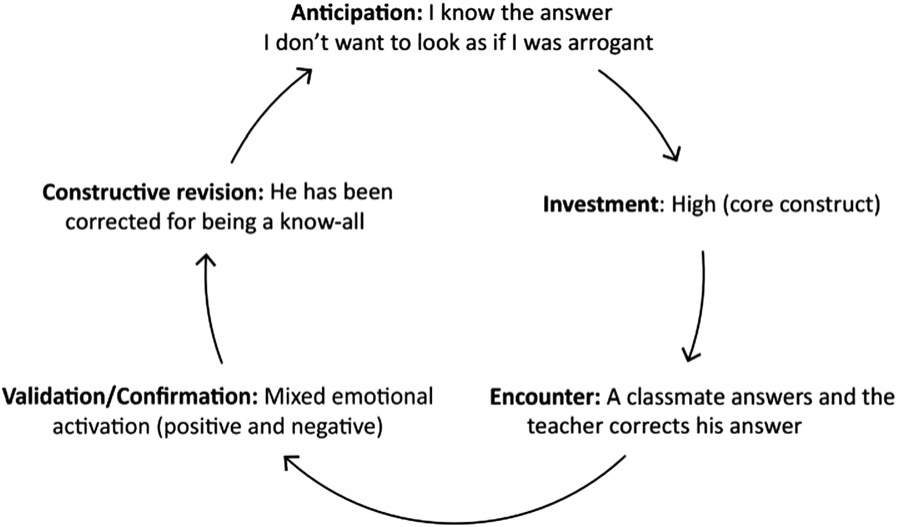
Example of Cycle of Experience involving an Implicative Dilemma. In this example, the Implicative Dilemma is formed by the congruent construct “humble-arrogant” and the discrepant construct “shy-sociable”. The hypothetical situation is as follows: The patient is attending a class and the teacher poses a question for the students to provide an answer. The patient knows the answer and experiences a high level of anxiety

Whenever possible, after the exploration of the problematic situation, a cycle of experience schematic is drawn jointly with patients (see Fig. 6). This diagram helps in the understanding of how their anticipations are related to the way they experience the situation. It is recommended to work on the cycle of experience schema once the exploration of the whole episode is done and we have sufficient information.

At the end of the session we propose as a homework task for the patient to observe other situations where the dilemma manifests itself, observing his or her thoughts, emotions and acts as they were analyzed during the session.

#### Session 3: troubleshooting

1. The therapist has difficulties connecting the dilemma with the daily or conflictual situations the patient talks about. Firstly, it is necessary to bear in mind that not all problematic situations will be related to the dilemma. Thus, it is important to remind the patient of the objective of these sessions (agreed on in the first session of the module) and to ask explicitly for situations linked to the dilemma. On other occasions, the situations being explained ARE linked to the dilemma, although it may not appear so at first sight. In these cases, the therapist must explore the implications these situations have. To this aim it is especially useful to consider the laddering up done in the previous session and use the more superordinate constructs for making sense of the situation.
2. The patient explains multiple problems that happened during the week and the therapist doesn’t manage to focus the session towards the established task. The patient is asked about the situation(s) he or she provided with respect to its (their) relevance for the dilemma, reminding the patient of the objective agreed on in the first session. The patient is also reminded of the briefness of the treatment and the importance to not alter the objectives in order to be able to accomplish them. After this, the situation considered more relevant to work on in session is selected. If the patient prefers to work on those problems which are not related to the dilemma (for example, *“this thing that happened to me doesn’t have to do with the dilemma… but it is much more important”*) and if after doing that he or she is still unable to connect the work done with the dilemma which was agreed to be the focus of this treatment unit, the use of this manual should be discontinued.

#### Session 4: relational implications of the dilemma

The aim of this session is to explore together with the patient how his or her personal relations influence their way of constructing reality, especially in relation to the dilemma, and how one could generate alternative constructions more independently of these figures. To accomplish this objective, the relational implications of the dilemma are explored following the subsequent steps:

- Asking the patient to describe in detail a recent episode in which the dilemma was highlighted.
- Asking the patient to describe the people participating in this episode according to the constructs implicated in one of the dilemmas being worked on, paying special attention to the people who, more or less explicitly, are blocking the change (for example, warning the patient of the negative consequences of change). If it is considered acceptable to the patient, these people could be called “dilemma accomplices”. The patient is asked the weight he or she believes they have in the maintenance of his or her dilemmatic construction, and if he or she would like this to change. If that is so, would this change regarding these people facilitate the patient encountering alternatives or solutions to the dilemma?
- Next, people who can be considered an exception to the dilemma are identified in the grid of the patient, that is to say, those who simultaneously occupy the desired and congruent pole (eg those who are “sociable” and “humble”). If there are no such people in the grid, the patient is asked if he or she knows someone who might be so (even if it is a historical figure or a person of current fame). The therapist explores jointly with the patient how these people might respond in that situation. The idea is to generate alternative constructions to those of the dilemma which might help to cope with the situation in a different way.

*Example 1*

- P: … it was strange because it was a day I was feeling better. I woke up earlier and even put on make-up. My husband was very happy when he saw me, he said I looked pretty… he also said if I was going to go out and flirt around now… I don’t know, he said it jokingly… but it made me feel bad…
- T: What did you feel in that moment?
- P: That maybe I’d overdone it… maybe I shouldn’t wear make-up on a normal day, maybe it was too much.
- T. As if you had changed too much?
- P: Yes, something like that. Like I was vain.
- T: Do you think this has something to do with the dilemma we’re working on?

*Example 2*

- T: What do you think Maria would have said (exception to the dilemma) if she had seen you wearing make-up on a regular day?
- P: She would have been very glad! She always tells me I’m pretty, that I should dress up more often.
- T: Do you think that somehow she might have seen you as being “vain”?
- P: No! She always tells me I’m not ostentatious at all…

As a continuation to this exploration, the therapist asks the patient if he or she would like to make some changes in the way they interact with the people who, in one way or another, validate the dilemmatic construction in question. Alternatively, the therapist might ask if he or she is inspired to do so by the attitude of the people who represent an exception to the dilemma.

At the end of the session, as a homework assignment the therapist asks the patient to write the chapters that would constitute her or his life’s history, explaining that they will be worked on in the next session, which will be dedicated to the historical reconstruction of the dilemma. The therapist explains the homework assignment in more or less these words: “Next time we will work in the session with the story of your life, and the role this dilemma has had in your life. To make this work easier I would like you to put in writing the different chapters that, in your opinion, would make up your life. Think as if you were to write your autobiography and had to divide the story into chapters. Follow the criteria you consider: important events, more or less definite periods (for example school, university). However you wish. You simply have to put a title to every chapter, which captures the essence of that period. I would also like you to include a future chapter… The chapter you would like to write from now on, and also give it a title. Does that seem ok to you?”

#### Session 4: troubleshooting

1. The patient is very isolated, does not interact with anyone. In this case we can use past episodes in which the dilemma manifested itself with significant people (for example, family).
2. The patient has persistent problems with his or her couple or primary support group. These situations can produce an intense blockage in the change in the patient because, often, vicious circles appear which are very difficult to break. In these cases a more detailed analysis of the relational implications of the dilemma is necessary, and thus, when this problem appears, the “wildcard” session is dedicated to further the analysis (thus dedicating two sessions to the relational implications of the dilemma). It is important to highlight that the aim is not to strictly work on, for example, the couple problems, but rather to understand how these problems manifest themselves and influence the maintenance of the dilemma.

#### Session 5: historical reconstruction of the dilemma

The aim of this session is to understand the patient’s construct system at different moments of his or her history, and its coherence through time, identifying in time when the dilemma appeared and the different ways of experiencing it throughout the years. In this way, we aim to foster a historical sense of coherence. The goal now is, therefore, to continue with the elaboration of the dilemma with a historical perspective of its genesis and evolution, following the steps described below. Although for didactical purposes we show a sketch of the sequence of exploration, the issues should be addressed conversationally avoiding an interrogative style. The aim is to establish a meaningful dialogue with the patient where the presence and relevance of the dilemma in his or her history is explored:

- Review of the chapters of life which the patient brings. We ask the patient to explain briefly: Why have you titled them in this particular way? What do they mean in your life?, etc. The last chapter corresponds to the future, and is reserved to be commented on at the end of this process.
- For each of the chapters, we ask the patient to position him/herself regarding the constructs implicated in the dilemma. We pay special attention to the changes produced in this positioning. We explore, for example, if the position of the self on the discrepant construct has varied at any time, or if the now dilemmatic construct was not so in the past, tending clearly to one of the poles in some situations.
- Gathering the information obtained in previous sessions, we also explore the position the “dilemma accomplices” have occupied progressively for the explored dilemmas and how they have reacted to the variations the patient has experienced throughout his or her history.
- Finally, the chapter dedicated to the future of life without the dilemma is reviewed. This review is done without going into details (which will be worked on in the session dedicated to future projection). The aim is to observe the coherence of this chapter within the patient’s life story. The issues to consider here are, mainly, whether the future chapter results from a personal evolution (or rather is a kind of magical or idealized leap), whether it makes sense within the patient’s history, and whether it integrates the relevant issues of that history.

*Example 1*

- T: You dedicated the first chapter of this autobiography to your childhood. You named it “those wonderful years”. It seems it was a good period for you. How do you remember it?
- P: Calm, it was a calm period. I was with my parents and siblings… I played with my brothers often, and our parents left us to our own devices, mostly…
- T: I don’t know if in that period it was already important whether you were shy or sociable…
- P: Well, it was different, because I have always been shy, but it didn’t worry me then. I was a bit shy and that was all, I also had friends.
- T: Would you say your parents saw you as a shy person? What did they say to you?

*Example 2*

- T: Afterwards it comes the chapter entitled “beetroot”. How do you remember that time?
- P: Terrible, that was when my problem really started, because I felt much shier with everything, and it was the time girls went out with boys… I wanted to be more sociable, not so shy.
- T: At that time, how did you feel in regard to the other traits we have talked about, like being humble?

At the end of the session, the proposed task is the writing, not of the patient’s history, but of the dilemma’s history. The therapist explains the homework assignment in more or less these words: “Continuing with this historical perspective, I would like to ask you to do a homework task which I believe will help us to continue understanding this dilemma and the importance it has had in your life… To resolve it! I would now like for you to write the history of your dilemma: how it appeared, how it has evolved, how you have experimented with it through time and how the important people in your life have. What do you think about doing this task?”

#### Session 5: troubleshooting

1. The patient presents a history of abuse, bereavement or trauma. This treatment manual focuses exclusively on the elaboration of personal dilemmas. For this reason, when a patient presents a history of this type the therapist must remember the treatment goals mutually agreed on in the first session. Notwithstanding, these problems tend to affect very important aspects of the patient’s life, and must not be left aside. Thus, the therapist must explore these aspects associated to trauma, focusing on how they have influenced the genesis and evolution of the dilemma. The resources mobilized by the patient to get through these traumatic situations are also explored (to the extent to which they were used) in order to generalize them to the resolution of the dilemma. When these situations exist in the history of the patient, it is recommended to dedicate the “wildcard” session to work with them.
2. The therapist does not feel comfortable focusing on the work on dilemmas in the face of a history of abuse, negligence or trauma by the patient. When faced with stories of abuse, bereavement or trauma, the therapist may feel uncomfortable working on aspects that can be seen as more peripheral. It is necessary to recall the brief format of the treatment module and the impossibility, on these occasions, of a more detailed work with this history of trauma. The available time is dedicated to the exploration of resources so as to generalize them to the change that is trying to be promoted in the treatment module.

#### Session 6: integration of the dilemma

The aim of this session is to clarify both parts of the dilemma at the end of the treatment module to accomplish an integration of both, an agreement. At the beginning of the session, the homework task is revised: the history of the dilemma. We explore alongside the patient how he or she felt when writing it, what he or she reflected, etc., and the end of the story is linked to the objective of the last session: to explore life without the dilemma.

Next, in order to accomplish the specific objective of this session, the two-chair dialog technique is used, in order to develop an interplay between the two parts of the self of the patient which constitute the dilemma (the part that wishes to change and the one that does not want to do so or is afraid to do so). The subsequent steps are followed:

- The technique is presented to the patient, explaining what it consists of and its aim. In this presentation, it is important to clarify any doubts or reluctance on the patient’s part in regard to the technique. For that purpose, the following phrasing could be used: “In today’s session, I would like us to use a technique that is a little different to the ones we have used until now. It consists in establishing a dialog between the two parts of you, of your dilemma, so that each one can express what it feels and what it wants. In this way, we can search for an agreement between them. To establish this dialog, we are going to use two chairs that we will put opposite each another. On one of the chairs will be the part of you that wishes to change, the one that wants to stop being an X person (for example, “shy” or “depressed”). On the other chair the part of you that would rather not change so as to not become a Y person (for example, “arrogant” or “selfish”). In this exercise you will not have to talk with me, rather directly to the other part of you, the one that will be in the other chair. Does that seem alright to you? Do you think it could be useful? Do you have any questions?”
- The patient sits in the chair of “the change”. The therapist must help him/her visualize in the chair in front of him/her the other part of her or himself, the one that does not wish to change, imagining how she or he is dressed, how she or he is sitting in the chair, what her or his posture is, etc. In this way differentiation of both parts is facilitated, and the patient talks from each of them according to her or his position.
- Next, the therapist asks the patient to explain to the other part of him or herself why it is valuable to change: the advantages of the change, the disadvantages of not changing, etc. The patient is reminded he or she must address the other part directly and try not to talk to the therapist.
- In the next step, the patient is asked to change seats, and from the part of him/herself that does not want to change, explain why this change is not advisable, and what risks it may entail. The patient is asked, if possible, to respond to the arguments the other part has used from the other chair. If necessary, the patient is reminded again to talk to the other part of her or himself rather than to the therapist.
- From this moment on, the therapist must facilitate a flowing dialog between the two parts, in which each part expresses what it wants or needs. To this end the patient is asked to change seats as many times as necessary. The dialog must flow towards a point in which each part expresses a petition to the other part and a satisfactory agreement, which respects both parts’ needs, is searched for.

Some examples of the phrasing of the above mentioned indications could be:

“Now you are seated in the chair of change. In front of you is the part of you that does not wish to change which, as we have seen in these sessions, would rather continue being X (for example, “shy” or “depressed”) to not risk turning into someone Y (for example, “arrogant” or “selfish”). I would like you to imagine how this part would be seated in front of you, what her or his posture is like, how she or he looks at you, even, how she or he’s dressed! Can you imagine it?”

“Could you explain to the other part why you would like to change?”

“What advantages does this change have for you?”

“What would the disadvantages of not changing be?”

“If you could ask this other part of you one thing that you do not wish to change, what would it be?”

“How could you reassure him/her about the change you want to make?”

“How could you assure him/her that you will not turn into a Y person (for example, arrogant or selfish)?”

“Could you explain to him/her why it would not be good to change?”

“What worries you if P (name of the patient) manages to turn into a Z person (for example, sociable or happy)? What could happen?”

“If you could ask something of this part of you that wants to change, what would you ask?”

“What could that part do to reassure you about the appropriateness of change?”

“How would you be sure that she or he doesn’t turn into someone Y (for example, arrogant or selfish)?”

These questions are merely indicative to guide the process of the dialog. The therapist can modify their order, reframe them, include new ones, etc. The desired objective is to reach an agreement between both parts of the dilemma.

#### Session 6: troubleshooting

The patient is reluctant about the technique, due to embarrassment, performance anxiety, feelings of ridicule, etc. In the first place, the therapist must reassure the patient regarding the technique: it is merely another exercise, even if it takes the shape of something different; the patient must not be troubled with “doing it right” or with knowing how to act or represent something, the important thing is that it be useful. In the second place, if the person continues to be uncomfortable with the technique’s format, the necessary adjustments for her or him to be comfortable can be arranged, for example, changing chairs but instead of talking to him/herself, talking to the therapist.

#### Session 7: future projection: living without the dilemma

The aim of this session is to generate an image of future in the patient’s life, in which the dilemma is resolved in a coherent way with his or her sense of identity. To accomplish this objective the patient is asked to imagine how his or her life will be when the dilemma is resolved (without specifying in which direction the dilemma will be resolved). If changes in this aspect have already begun to appear, the question is adapted and directed to explore the extent to which the change has been established, its implications in various aspects of the patient’s life (especially in his or her family or intimate relationships) and the contrast with life previously.

7.1 Example with an implicative dilemma
  “Remember that magic wand (therapist shows pencil) we talked about in the first session? When I asked you if you were sure of becoming a (desirable pole of the discrepant construct) person? If now, with this wand, I could quickly solve this dilemma we have been talking about, what do you think your life would be like? If I were to touch you with my magic wand and that would be it, you no longer have a dilemma… What would your life be like from the moment you step out through that door?”
7.2 Example with a dilemmatic construct
  “Imagine this is a magic wand (shows a pencil) and that if I were to touch you with it the dilemma we have been talking about these sessions would resolve itself in an instant. What do you think your life would be like? If I were to touch you right now with my wand and that would be it, no more dilemma… What would your life be like from the moment you step out through that door?”

From this introduction a significant dialog is generated where the therapist, through questions, helps the patient detail how his or her life would be without the dilemma. The said dialog must not be in an interrogative style, more as a conversation where the therapist is genuinely interested by the diverse manifestations and implications of the change. The questions may be more or less with the following words:

“What would you notice first once the dilemma is resolved?”

“What things will you do differently?”

“What will other important people to you (your partner, father, mother) notice?”

“How do you think these people will react regarding the dilemma’s resolution?”

“What things will remain the same, won’t change?”

It is not necessary to respect this sequence of the questions, which are shown merely as an example, and the therapist may add the questions he or she considers necessary to adequately capture life without the dilemma.

Once an image of the future is accomplished with the dilemma being resolved, we explore which aspects of life without the dilemma are already happening. Next, we explore how to extend or generalize these exceptions to other moments and contexts, in more or less these words:

“From all of these changes we have been talking about, which do you think have already begun?”

“I would like us to focus on these changes; it does not matter if they are small changes.”

“What has happened?”

“How did you feel?”

“How did X (your partner, mother, father) react?”

“What would have to occur for it to happen again?”

“In which other situations could this change occur again?”

“What should you or someone else do to reverse change and return to the problematic situation?”

The sequence that the questions follow is not compulsory, it is shown merely as an example, and the therapist may add the questions he or she considers necessary to make the characteristics of life without the dilemma clearly visible in the patient’s everyday and immediate reality.

#### Session 7: troubleshooting

The patient has difficulty imagining what life without the dilemma would be like. Occasionally, people live with symptoms for years and find it very difficult to imagine themselves without this problem. In these situations the therapist can dedicate the “wildcard” session to working on this aspect. Then, the patient is asked to reflect on this idea during the week, and if he considers it necessary and convenient, he may ask people close to him about this issue. For example, the patient could thus initiate a dialog on the subject: *“If suddenly I were to change and I was a much happier person, but I still remained myself… that is to say, without changing in other important aspects of myself… what do you think I would do differently?”*

## Discussion

In the last three decades therapy manuals have become a common and necessary ingredient of controlled research in psychotherapeutic approaches. However, while approaches such as CBT have been prolific with producing and applying therapy manuals both in research and practice authors based on PCT have produced very few therapy manuals which would then be included in RCTs. In this article a new therapy manual based on PCT is presented for the treatment of depression. Although it is not a full treatment manual (rather, it is a specific intervention), the fact that the manual has been produced and included in a controlled studied aimed to test its efficacy is a substantial step in the development, and potential growth, of constructivist approaches to psychotherapy.

PCT is a predecessor of many cognitive approaches to personality and psychotherapy but in its own development as a differentiated model it has retained, and strengthen some of its distinctive features. Among them, the notion that meaning systems are not logical systems (see fragmentation corollary [4]) has evolved into the exploration of internal conflicts within the cognitive system. Curiously, the notion of cognitive conflicts is not apparent or explicit in cognitive-behavioral therapies in which the emphasis is on cognitive distortions or maladaptive beliefs. What the notion of cognitive conflicts embraced in this work suggests is that some core beliefs (“core constructs” in PCT) rather than being dysfunctional per se they may be in conflict among them and, thus, generating a dilemma for the patient who is contemplating change and improvement. Therefore, the main advantage of the manual presented here is that it describes a systematic procedure based on the RGT to detect cognitive conflicts and puts forward a series of techniques aimed to assist patients in the resolution of their often implicit, personal dilemmas [75].

The DFI presented here in the format of therapy manual, following guidelines which have been proposed for these manuals [3], is that now its efficacy can be empirically tested. To this aim, the manual is reasonably brief, limited to eight sessions, and all the procedure and techniques are described in detail and with case vignettes to facilitate its implementation. In the study in which it has been included [2], DFI is compared to CBT (both in individual therapy format) for patients with a diagnosis of unipolar depression who have participated in seven group CBT sessions before being randomized to either DFI or CBT. Thus, having the manual for the DFI permitted to test the efficacy of the therapy work presented here focused on dilemma resolution [75] rather than on restructuring maladaptive beliefs. And, more important, this manual will enable replication studies and will facilitate dissemination of this psychological intervention among professionals. Results of the efficacy study provide preliminary support [76] of the usefulness of DFI for depression as an ingredient of a more comprehensive cognitive-behavioral therapy. In any case, the mere existence of the manual offers therapeutic alternatives for intervention which can be included in a therapy process as a function of its efficacy or the appropriateness of the approach for a particular patient. That is, DFI expands the range of psychotherapeutic choice in the treatment of depression. And the addition of therapeutic alternatives opens an opportunity for treatment improvement in depression.

A more controversial issue is whether the DFI manual presented here can be used also in the context of therapy approaches other than CBT. While the fact of having described in a detailed manner all the therapeutic procedures might facilitate the inclusion of DFI in other existing treatments, its actual inclusion cannot be taken as granted. Rather, it must be carefully studied as a function of the treatment in which it is being considered for inclusion. Further studies with a research design similar to that of the current one [2] should be carried out to estimate the usefulness of such an inclusion.

Probably, one of the major limitations of the DFI manual presented here is the level of expertise needed for both the administration of the RGT (and the data analysis leading to the identification of the cognitive conflicts of the patient) and for the application of the techniques described in the manual. Although the RGT has been described in various publications [18, 19, 21] and the therapy manual is, as said, detailed and with examples some specific training is surely needed to perform them with some degree of competence and reliability. The cost of this additional training might discourage some researchers form including DFI in their studies and professionals from adopting dilemma work in their everyday practice. Still, some might find it stimulating to invest in some training in order to be able to increase the range of their therapeutic alternatives.

## Conclusions

Therapy manuals are an essential ingredient of controlled studies aimed to provide evidence for the efficacy of psychotherapeutic interventions. The manual presented here has been created following well-recognized guidelines for manual development for inclusion in randomized clinical trials aimed to improve the treatment outcome for depression. The present Dilemma-Focused Intervention manual has been used in one of such studies in combination with group cognitive-behavioral therapy [2] with some supporting evidence [76]. Thus, we can consider this manual as a practical addition to the existing repertoire of interventions for the research and practice of the psychotherapy for depression. Further studies are needed to verify the efficacy of DFI and to validate it as an efficacious alternative treatment ingredient.

## Abbreviations

CAT, cognitive analytic therapy; CBT, cognitive-behavioral therapy; DFI, dilemma-focused intervention; PCT, personal construct theory; RGT, repertory grid technique
